# Air pollution and DNA methylation alterations in lung cancer: A systematic and comparative study

**DOI:** 10.18632/oncotarget.13622

**Published:** 2016-11-25

**Authors:** Cheng-Lan Jiang, Shui-Wang He, Yun-Dong Zhang, He-Xian Duan, Tao Huang, Yun-Chao Huang, Gao-Feng Li, Ping Wang, Li-Ju Ma, Guang-Biao Zhou, Yi Cao

**Affiliations:** ^1^ Laboratory of Molecular and Experimental Pathology, Kunming Institute of Zoology, Chinese Academy of Sciences, Kunming 650223, China; ^2^ Kunming College of Life Sciences, University of Chinese Academy of Sciences, Kunming 650223, China; ^3^ School of Life Sciences, University of Science and Technology of China, Hefei 230026, China; ^4^ Institute of Health Sciences, Shanghai Institutes for Biological Sciences, Chinese Academy of Sciences, Shanghai 200031, China; ^5^ Department of Thoracic and Cardiovascular Surgery, The Third Affiliated Hospital of Kunming Medical University, (Yunnan Tumor Hospital), Kunming 650106, China; ^6^ Department of Thoracic Surgery, The Third Affiliated Hospital of Kunming Medical University, (Yunnan Tumor Hospital), Kunming 650106, China; ^7^ Department of Thoracic Surgery, The First People's Hospital of Yunnan Province, Kunming 650032, China; ^8^ Clinical Medicine Research Center, The First Affiliated Hospital of Kunming Medical University, Kunming 650332, China; ^9^ State Key Laboratory of Membrane Biology, Institute of Zoology, Chinese Academy of Sciences, Beijing 100101, China

**Keywords:** air pollution-related lung cancer, DNA methylation, benzo(a)pyrene, carcinogenesis, vitamin

## Abstract

The lung cancer incidence in the Xuanwei and neighboring region, Yunnan, China, is among the highest in China and is attributed to severe air pollution with high benzo(a)pyrene levels. We systematically and comparatively analyzed DNA methylation alterations at genome and gene levels in Xuanwei lung cancer tissues and cell lines, as well as benzo(a)pyrene-treated cells and mouse samples. We obtained a comprehensive dataset of genome-wide cytosine-phosphate-guanine island methylation in air pollution-related lung cancer samples. Benzo(a)pyrene exposure induced multiple alterations in DNA methylation and in mRNA expressions of DNA methyltransferases and ten-11 translocation proteins; these alterations partially occurred in Xuanwei lung cancer. Furthermore, benzo(a)pyrene-induced DKK2 and EN1 promoter hypermethylation and LPAR2 promoter hypomethylation led to down-regulation and up-regulation of the genes, respectively; the down-regulation of DKK2 and EN1 promoted the cellular proliferation. Thus, DNA methylation alterations induced by benzo(a)pyrene contribute partially to abnormal DNA methylation in air pollution-related lung cancer, and these DNA methylation alterations may affect the development and progression of lung cancer. Additionally, vitamin C and B6 can reduce benzo(a)pyrene-induced DNA methylation alterations and may be used as chemopreventive agents for air pollution-related lung cancer.

## INTRODUCTION

Diseases caused by air pollution have become a serious health problem worldwide. For example, approximately 350,000–500,000 people in China die prematurely each year as a result of air pollution [1]. Because the human respiratory system is open, carcinogens that are present in polluted air can come into direct contact with and attack lung epithelial cells. Numerous studies have demonstrated a strong link between air pollution and lung cancer. If lung cancer in non-smokers was considered as a separate disease, this malignancy would be ranked the seventh most deadly cancer [2]. Air pollution causes more than 200,000 lung cancer deaths globally (Global burden of disease pattern, 2010. Available at: http://www.healthdata.org/notice-tool-migration). The International Agency for Research on Cancer (IARC) has classified air pollution as a Group 1 carcinogen (carcinogenic to humans) [3]. The risk of lung cancer correlates with average pollution levels [4, 5], and the morbidity and mortality of air pollution-related lung cancer vary significantly across different regions with high rates of air pollution-related lung cancer often occurring in areas with high air pollution. The cases of lung cancer in these areas represent good models for studying the relationship between environmental factors and this fatal disease. Examples of air pollution-related lung cancer exist in Xuanwei City and neighboring region (Fuyuan County), Yunnan Province, China [6–8]. In these districts, the lung cancer incidence is four to five times higher than the national average, and non-smoking women suffer from lung cancer more frequently compared to those in other areas. The high risk of Xuanwei and Fuyuan lung cancer (XWLC) is attributed to exposure to indoor and outdoor air pollution caused by burning smoky coal. When smoky coal is burned, high concentrations of cancer-causing substances such as polycyclic aromatic hydrocarbons (PAHs), are released [9]. The polluted air in these districts contains higher concentrations of PAHs than in other areas [10]. PAHs are highly carcinogenic and are key carcinogens of XWLC. Benzo(a)pyren (BaP) is the most common PAH, and BaP exposure levels are strongly associated with the incidence of XWLC (Supplementary Figure S1). In these highly air-polluted regions, the primary source of BaP exposure could be attributed to burning coal in the home, and smokers had slightly higher BaP exposure than non-smokers [11]. The findings in Xuanwei have been cited in the IARC monograph (World Health Organization IARC, 2010). Our previous studies demonstrated that XWLCs showed some distinct characteristics at the molecular level compared with lung cancers in other regions [11–13].

Carcinogenesis is a multi-factor and multi-stage process. Epigenetics also participates in carcinogenesis [14–18]. DNA methylation, which primarily refers to the methylation of the 5-carbon on cytosine residues (5-mC) in cytosine-phosphate-guanine (CpG) dinucleotides, is one of the most important epigenetic marks. Altered DNA methylation is common in lung cancer [19–25], and environmental pollution can cause DNA methylation changes [26–34]. PAHs that are produced by burning coal, gasoline, diesel, and tobacco, are key environmental carcinogens associated with air pollution-related lung cancer, including XWLC. BaP is the best index for estimating the level of PAHs. In the present study, we performed genomic methylation analyses in XWLC tissues, XWLC cell lines, and BaP-treated cells and animals to characterize genomic methylation profiles of air pollution-related lung cancer and to study the relationships among DNA methylation, BaP exposure, and air pollution-related lung cancer. Furthermore, the functions and mechanisms of DNA methylation induced by BaP exposure were investigated. Cancer prevention is extremely valuable, and the use of chemopreventive drugs is one possible strategy to prevent cancer. Vitamins participate in the regulation of DNA methylation [35–37], therefore, we also examined whether vitamin could influence BaP-induced DNA methylation alterations.

## RESULTS

### Genomic methylation profiles in XWLC tissues

**Figure 1:**
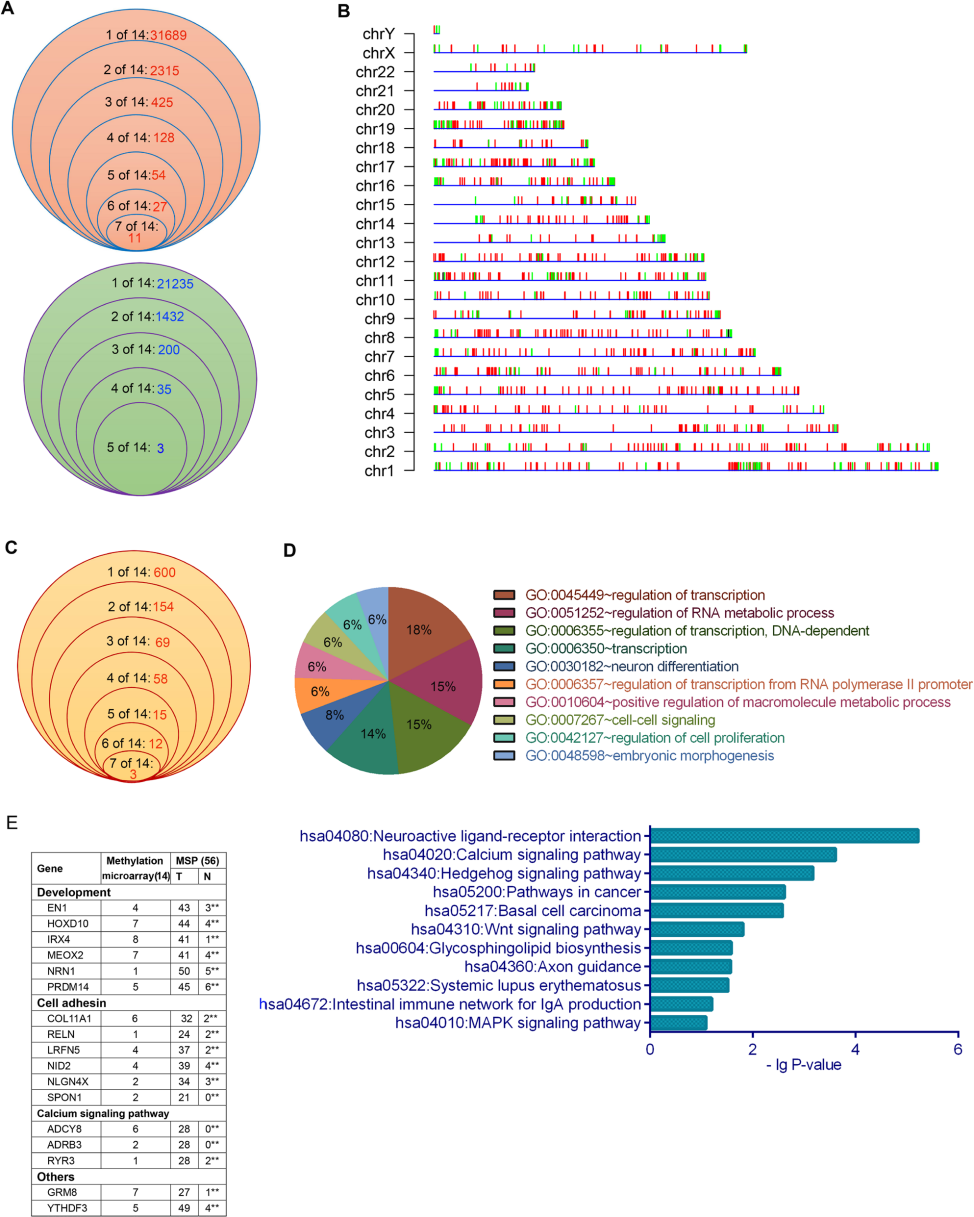
DNA methylation profiling in XWLC tissues **A.** The frequency of hypermethylated (above) and hypomethylated (below) regions with a peak score ≥ 2 and |log2 ratio| > 0 in the 14 XWLC tissues. **B.** Chromosomal distribution of DMRs with a peak score ≥ 2 and |log2 ratio| > 1 in the 14 XWLC tissues. Red and green vertical lines represent hypermethylated and hypomethylated regions, respectively. **C.** The frequency of genes whose promoters contained DMRs with a log2 ratio > 1 in the 14 XWLC tissues. **D.** GO and KEGG analyses of genes whose promoters contained DMRs with a log2 ratio > 1. **E.** Methylation analysis of the 17 selected genes using MSP in the 56 paired XWLC tissues, **P < 0.01 (chi-square test).

The present study used fourteen XWLC tissue samples that had been identified as non-small cell lung cancer (NSCLC) and included 11 cases of adenocarcinoma (AD) and three cases of squamous cell carcinoma (SCC); eight out of the 14 patients were the never smoking women; the 14 cases of XWLC were used for whole genome sequencing in a previous study [11]. In the present study, the 14 XWLC and paracancerous tissues were analyzed for genomic methylation using microarrays (Supplementary Table S1A). The log2 ratios, which represented quantitative methylation levels of differentially methylated regions (DMRs), ranged from 0 to 3.03 for hypermethylated regions and from -0.0011 to -2.095 for hypomethylated regions. The 14 XWLCs exhibited heterogeneous levels and patterns of DNA methylation. The occurrence frequency of hypermethylated and hypomethylated regions in the 14 XWLCs is shown in Figure 1A and Supplementary Table S2. The DMRs were widely distributed in all chromosomes (Figure 1B).

Interestingly, the 911 hypermethylated and 225 hypomethylated regions with | log2 ratio | > 1 occurred at gene promoters. The occurrence frequency of promoter-hypermethylated and promoter-hypomethylated genes in the 14 XWLCs is shown in Figure 1C and Supplementary Table S3. The promoter-hypermethylated genes were analyzed using Gene Ontology (GO) and Kyoto Encyclopedia of Genes and Genomes (KEGG). These genes were primarily associated with neuroactive ligand-receptor interaction, Wnt signal pathways, calcium signal pathways, development, cell adhesion, and cell proliferation, among others (Figure 1D).

To validate the microarray data, we selected 17 genes with 17 DMRs to analyze the methylation of specific sites using methylation-specific polymerase chain reaction (MSP) in 56 lung cancer tissues (Supplementary Table S1A). The 16 selected hypermethylated regions were all located at promoters, with the exception of one hypermethylated region at the left side of YTHDF3 promoter. MSP results were consistent with the microarray data. There were significant differences in the occurrence of the 17 DMRs between cancerous and paracancerous tissues (Figure 1E). Interestingly, over 80% of lung cancer specimens contained hypermethylated regions within the promoters of NRN1 and PRDM14 and at the left side of the YTHDF3 promoter. Moreover, the relationships between the methylation statuses of the 17 DMRs and clinicopathologic characteristics were analyzed in 56 patients (Supplementary Table S4).

### Genomic methylation profiles in lung cancer cell lines

Genomic methylation profiles were analyzed using microarrays in five cell lines, including immortalized human bronchial epithelial cells (IHBECs; 16HBE), lung AD (A549), lung SCC (EPLC-32M1), and XWLC (XLA-07 and XL-JT; both are AD). Compared to 16HBE cells, there were 75,006, 54,077, 46,484, and 35,528 hypermethylated sites in XLA-07, XL-JT, EPLC-32M1 and A549 cells, respectively. Additionally, 23,898, 29,980, 83,528, and 40,221 hypomethylated sites occurred in XLA-07, XL-JT, EPLC-32M1, and A549 cells, respectively. Differentially methylated sites (DMSs) that appeared in the four lung cancer cell lines are shown in Supplementary Table S5.

### Genomic methylation profiles in BaP-exposed culture cells

**Figure 2:**
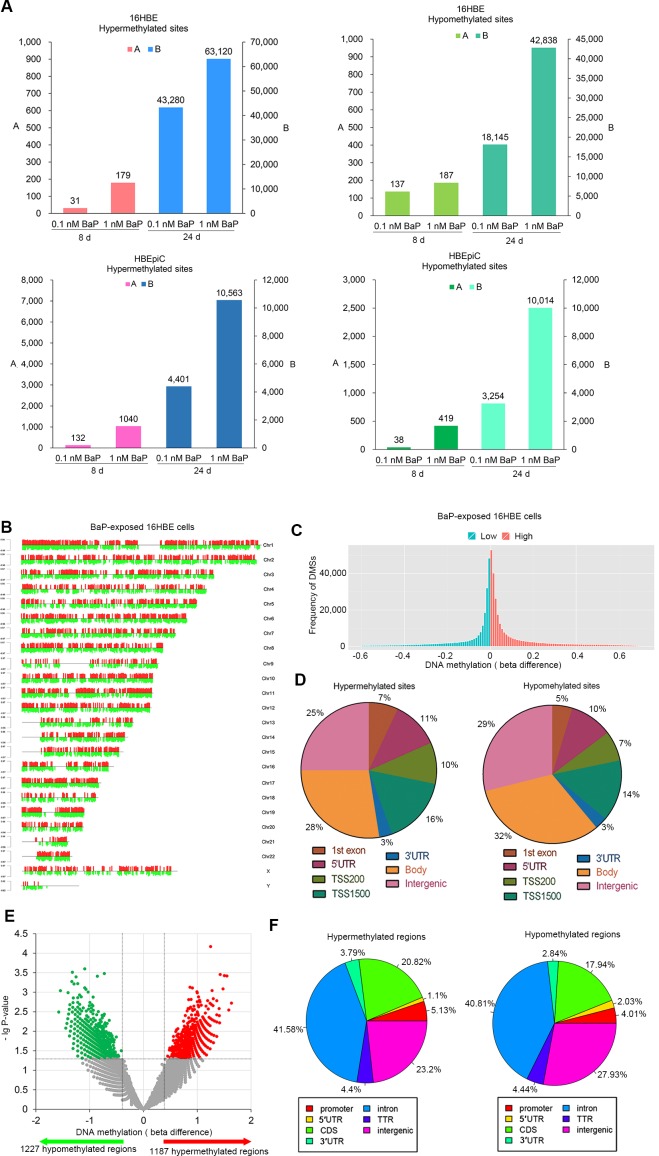
DNA methylation profiling in BaP-exposed IHBECs and murine tissues **A.** DMSs in BaP-exposed 16HBE and HBEpiC cells. DMSs with |beta difference| > 0.1 for IHBECs treated with BaP for 8 days; DMSs with |beta difference| > 0.2 for IHBECs treated with BaP for 24 days. **B.** Chromosomal distribution of DMSs with |beta difference| > 0.2 that occurred at least once in the four varieties of BaP-exposed 16HBE cells. Red and green vertical lines represented hypermethylated and hypomethylated sites, respectively, and the length of the vertical lines indicates methylation level. **C.** Normalized histogram of DMSs with |beta difference| > 0.2 shows total DNA methylation statuses in 16HBE cells treated with BaP at varying concentrations and different treatment times. Red and green represented hypermethylated and hypomethylated CpGs, respectively. **D.** Distribution of DMSs with |beta difference| > 0.2 at various functional regions of the genome in BaP-exposed 16HBE cells. **E.** Volcano plot of DMRs in BaP-exposed murine tissues. **F.** Distribution of DMRs at various functional regions of the genome in BaP-exposed murine tissues. 1st exon: gene first exon; 3′UTR: 3′-untranslated region; 5′UTR: 5′-untranslated region; TSS200: upstream promoters at -200 bp of transcription start site; TSS1500: upstream promoters at -1500 bp of transcription start site; Body: gene body; Intergenic: intergenic region (DNA sequences located between genes); CDS: gene coding sequence; TTR: downstream at 5000 bp of transcription termination site.

Genomic methylation profiles were analyzed in BaP-treated IHBECs (16HBE and HBEpiC cells) using microarrays. Compared with the solvent (dimethyl sulfoxide, DMSO) treatment, numerous DMSs were observed in BaP-treated IHBECs (Supplementary Table S6). In total, the number of DMSs increased with enhanced BaP-treated concentrations and prolonged BaP exposure (Figure 2A). The normalized histogram and heat-map analyses showed the same trend (Figure 2C and Supplementary Figure S2B, Supplementary Figure S3), and the DMSs existed in all chromosomes (Figure 2B and Supplementary Figure S2A). The distribution of DMSs is shown in Figure 2D. Thus, BaP can induce DNA methylation alterations *in vitro*, and the degree of alteration is positively related to BaP exposure levels.

### Genomic methylation profiles in BaP-exposed murine tissues

To confirm whether BaP exposure can affect DNA methylation *in vivo*, the BaP-treated murine skin was used for genomic methylation analysis using methylated DNA immunoprecipitation sequencing (MeDIP-Seq). Compared with the solvent control, a total of 2,414 DMRs were identified in BaP-treated samples (P < 0.05). In total, 1,187 and 1,227 regions exhibited hypermethylation and hypomethylation, respectively (Figure 2E and Supplementary Table S7). The DMR distribution is shown in Figure 2F. A total of 87 and 66 genes contained hypermethylated and hypomethylated regions at promoters, respectively; according to KEGG analysis, these genes are involved in several signaling pathways. Therefore, BaP can also induce DNA methylation alterations *in vivo.*

### Comparison of DMSs between lung cancer cell lines and BaP-exposed cells

**Figure 3:**
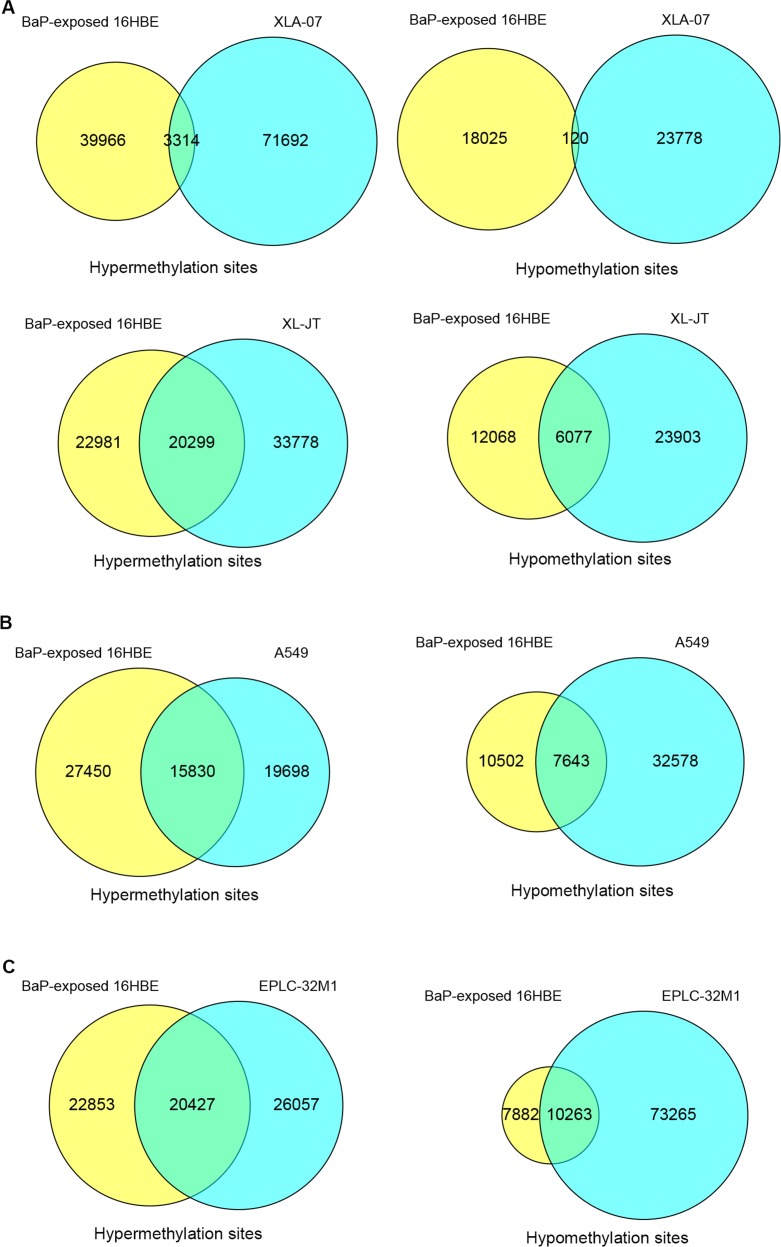
Comparison of DMSs between BaP-exposed IHBECs and lung cancer cell lines using a Venn diagram **A.** Overlaps of DMSs between XWLC cell lines (XLA-07 and XL-JT) and BaP-exposed 16HBE cells. **B.** Overlaps of DMSs between lung AD cell line (A549) and BaP-exposed 16HBE cells. **C.** Overlap of DMSs between a lung SCC cell line (EPLC-32M1) and BaP-exposed 16HBE cells. In A-C, 16HBE treated with 0.1 nM BaP for 24 days.

Some of the DMSs induced by BaP exposure existed in cultured lung cancer cells (Figure 3A-3C). The overlapping DMSs exhibited the following characteristics: 1) the hypermethylated and hypomethylated sites overlapped between BaP-exposed 16HBE cells and cultured lung cancer cells; 2) approximately 0.6% to 56% of DMSs detected in BaP-exposed 16HBE cells occurred in various lung cancer cell lines; 3) DMSs induced by BaP exposure existed in both AD and SCC. The overlapping DMSs of BaP-exposed IHBECs and cultured lung cancer cell lines are listed in Supplementary Table S8. DNA methylation alterations induced by BaP may partially contribute to abnormal DNA methylation, with varying degrees observed in both types of lung cancer.

### Comparison of global DNA methylation (5-mC) and hydroxymethylation (5-hmC) levels among BaP-exposed cells, XWLC cell lines, and XWLC tissues

**Figure 4:**
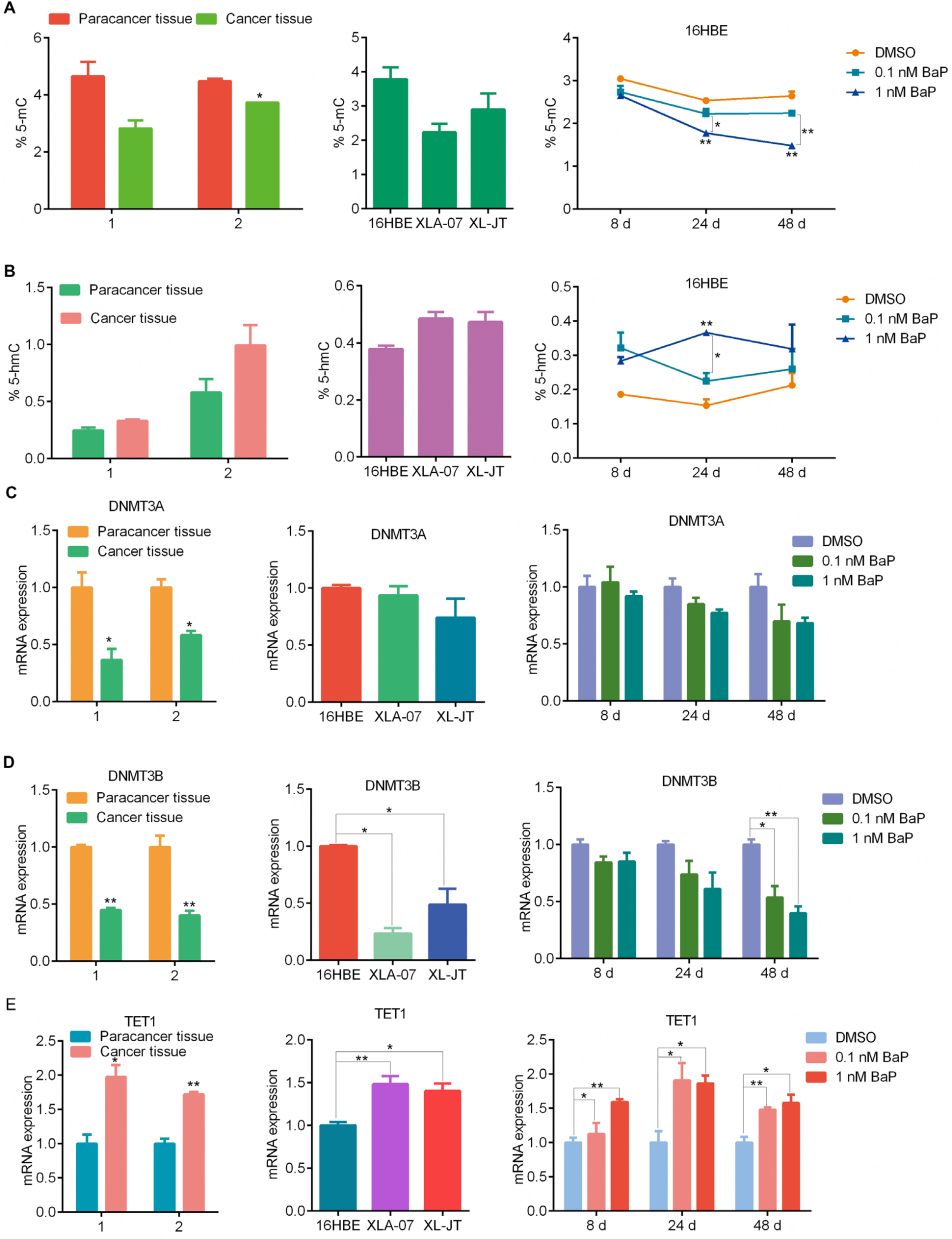
Comparison of 5-mC and 5-hmC levels, as well as DNMT3A, DNMT3B and TET1 mRNA expression levels 5-mC **(A)** and 5-hmC **(B)** levels were measured by ELISA in BaP-exposed 16HBE cells, XWLC cell lines, and paired XWLC tissues. DNMT3A **(C)** DNMT3B **(D)** and TET1 **(E)** mRNA levels were examined by qRT-PCR in BaP-exposed 16HBE cells, XWLC cell lines, and paired XWLC tissues. For BaP-exposed 16HBE cells, DMSO was used as the control. The results were analyzed using Student's t-test (**P < 0.01, *P < 0.05).

The level of 5-mC is reduced in cancer cells [18], DNA demethylation is associated with 5-hmC [38]. We examined global 5-mC and 5-hmC levels by solid-phase enzyme linked immunosorbent assay (ELISA). Compared to the control, global 5-mC levels were decreased and global 5-hmC levels were increased after BaP exposure (Figure 4A–4B). Similarly, the value of 5-mC was lower in XWLC cell lines than in 16HBE cells, and the value of 5-hmC was higher in the XWLC cell lines. Additionally, XWLC tissues showed lower levels of global 5-mC and higher levels of 5-hmC than did paracancerous tissues.

### Comparison of the expression of DNA methyltransferases (DNMTs) and ten-11 translocation proteins (TETs) among BaP-exposed cells, XWLC cell lines, and XWLC tissues

DNA methylation and demethylation are regulated by DNMTs and TETs [18, 38, 39]. We examined the mRNA expression levels of DNMTs and TETs using quantitative real-time polymerase chain reaction (qRT-PCR). The mRNA levels of DNMTs and TETs were altered in BaP-exposed 16HBE cells, however, the expression of various subtypes of DNMTs (DNMT1, DNMT3A, DNMT3B) and TETs (TET1, TET2, TET3) was different (Figure 4C-4E and Supplementary Figure S4). In cultured XWLC cells, various subtypes of DNMTs and TETs were found to have distinct expression patterns compared with 16HBE cells. Moreover, DNMT and TET mRNA expression was significantly altered in XWLC tissues compared to paracancerous tissues. We made the following observations: 1) the extent of changes in DNMT and TET mRNA expression was less in BaP-exposed cells than in XWLC cell lines and tissues, and 2) the expression patterns of DNMT and TET subtypes in BaP-exposed cells were partially similar to those in XWLC cell lines and tissues.

### Meta-analysis of DNA methylation and mRNA expression

First, we determined mRNA expression profiles in XLA-07, XL-JT, EPLC-32M1 and 16HBE cells by microarray analysis. Compared to 16HBE cells, 4937 and 5238 genes were up-regulated and down-regulated, respectively, in XLA-07 cells; 5342 and 3907 genes, in XL-JT cells; and 4547 and 3885 genes, in EPLC-32M1 cells. Additionally, 1047 up-regulated and 1117 down-regulated genes occurred commonly in all three cancer cell lines (Supplementary Table S9). Subsequently, relationships between DNA methylation and gene expression were investigated through meta-analysis according to the strategy shown in Supplementary Figure S5A.

**Figure 5:**
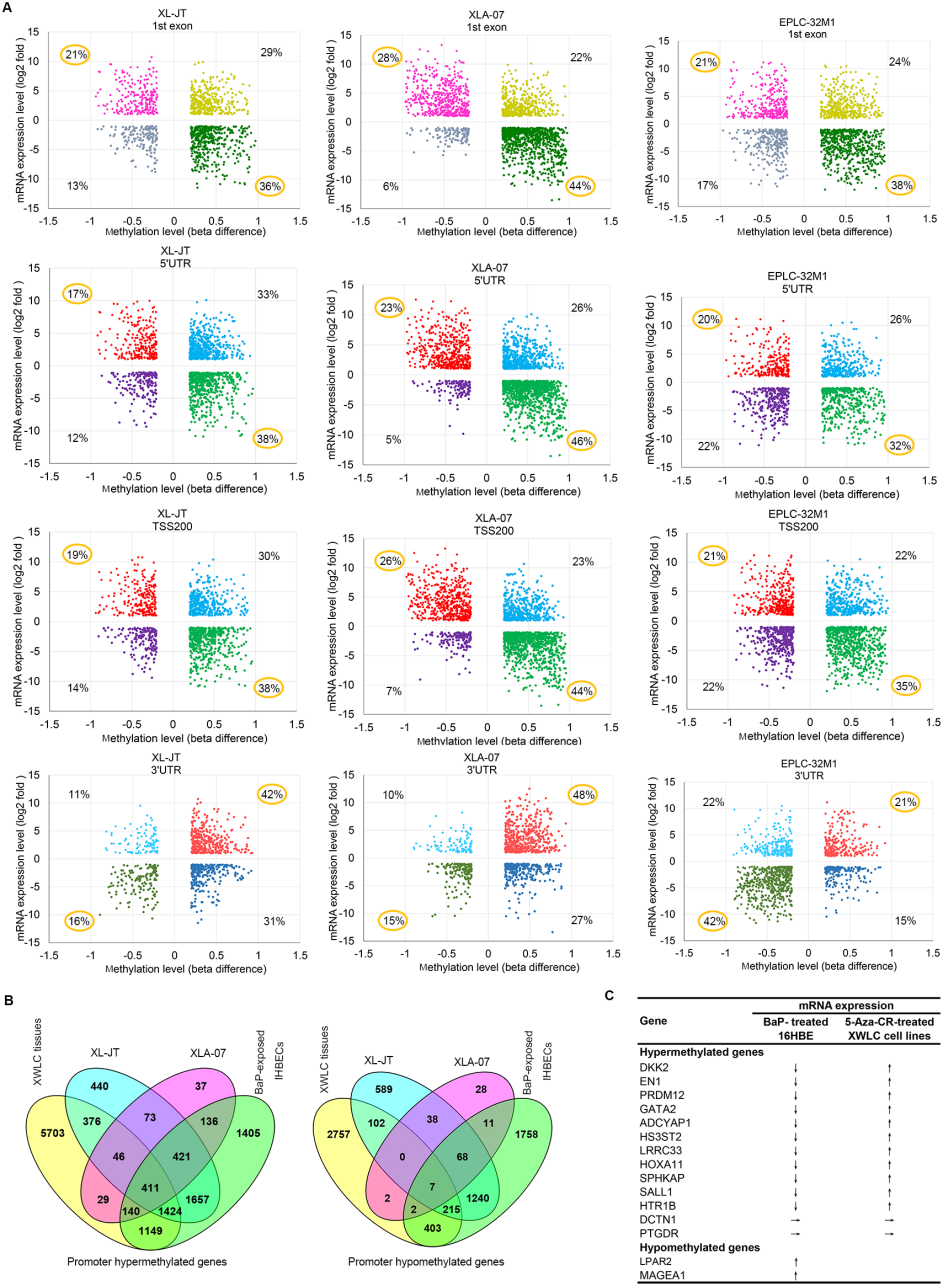
Relationships between DNA methylation and gene expression **A.** Starburst plot of genes showing the relationships between methylation levels (x-axis) at various regions (first exon, 5′UTR, TSS200, and 3′UTR) of genes and mRNA levels (y-axis) in lung cancer cell lines. **B.** Venn diagram showing overlap of promoter hypermethylated and hypomethylated genes among XWLC cell lines (XL-JT, XLA-07), XWLC tissues, and BaP-exposed IHBECs. In A and B, genes were selected according to the strategies described in Supplementary Figure 5A and 5C, respectively. **C.** mRNA expression levels of the 15 genes with differentially methylated promoters were changed in 16HBE cells treated with BaP and in cultured XWLC cells treated with 5-Aza-CR. ↓, ↑, and → represent down-regulation, up-regulation, and no change, respectively.

The meta-analysis revealed three cases: 1) DNA methylation levels were negatively correlated with mRNA expressions in more than 50% of genes (Figure 5A). In this case, DMSs occurred primarily around gene proximal promoter regions, including the first exon, 5′-untranslated region (5′UTR), and upstream promoters at -200 bp of transcription start site (TSS200). For examples, in XLA-07 cells, 72%, 69%, and 70% of genes possessed DMSs in the first exon, 5′UTR, and TSS200, respectively. For these genes, the hypermethylation and hypomethylation of these regions corresponded with down-regulation and up-regulation, respectively, of mRNA. 2) DNA methylation levels were positively correlated with mRNA expression in more than 50% of genes (Figure 5A). In this case, DMSs occurred primarily at the 3′-untranslated region (3′UTR). In XLA-07 cells, 63% of genes contained DMSs at their 3′UTR; the hypo- and hyper-methylation of the 3′UTR corresponded to the down- and up-regulation, respectively, of the mRNA expression of these genes. 3) Approximate 50% of the genes showed DNA methylation levels that were negatively correlated with mRNA expression levels, but the other 50% of the genes displayed DNA methylation levels that were positively correlated with mRNA expression levels (Supplementary Figure S5B). In this case, DMSs emerged primarily at the gene body and the upstream promoters at -1500 bp of transcription start site (TSS1500). Based on these observations, we estimated that the hypermethylation around promoter regions is associated with gene silencing and that hypomethylation is associated with gene transcription. In contrast, the increased methylation at the 3′UTR was related to increased gene expression and vice versa.

### Comparative analyses of promoter-hypermethylated and promoter-hypomethylated genes among XWLC tissues, XWLC cell lines, BaP-exposed IHBECs, and BaP-exposed murine tissues

Promoter methylation regulates gene expression [16, 18]. Our meta-analysis of DNA methylation and mRNA expression revealed that promoter methylation levels correlated with mRNA expression for a large proportion of differentially expressed genes in lung cancer. Here, we focused on the significance of promoter hypermethylation and hypomethylation induced by BaP. First, we compared genes possessing DMRs or DMSs at promoter regions, including TSS200, TSS1500 and 5′UTR, among XWLC tissues, XWLC cell lines, and BaP-exposed IHBECs using the VENNY tool (Supplementary Figure S5C). The integrated results are shown in Figure 5B and Supplementary Table S10. Subsequently, we compared the DNA methylation data of BaP-exposed murine tissues with those of human samples. In total, 87 promoter-hypermethylated genes and 66 promoter-hypomethylated genes were detected in BaP-exposed murine tissues. Orthologs of these genes in humans were identified using HomoloGene build 68. Thereafter, the VENNY tool was used to analyze differentially promoter-methylated genes among XWLC tissues, XWLC cell lines, and BaP-treated IHBECs and murine tissues (Supplementary Table S10). In total, 20 promoter-hypermethylated genes and 25 promoter-hypomethylated genes that were detected in BaP-exposed murine tissues also occurred in BaP-exposed IHBECs; the 30 and 7 genes in XWLC tissues.

### Relationships between promoter methylation and mRNA expression in selected genes

To confirm the relationships between promoter-methylation status and mRNA expression, 13 promoter-hypermethylated genes and two prompter-hypomethylated genes were selected through comparative analyses (Figure 5B). These genes were chosen according to the following parameters: appearing at least twice in 14 XWLC tissues with |log2 ratio| > 1; occurring in BaP-treated IHBECs with |beta difference| > 0.2; existing at least once in XWLC cell lines with |beta difference| > 0.2; and having important physiological functions and potential involvement in carcinogenesis. The mRNA expression levels of the 15 genes in BaP-exposed 16HBE cells are shown in Figure 5C. The mRNA expression levels of the 11 promoter-hypermethylated genes were decreased in BaP-exposed 16HBE cells compared to control cells. In contrast, the mRNA expression levels of the two promoter-hypomethylated genes (LPAR2 and MAGEA1) were increased in BaP-exposed 16HBE cells. Consistently, the mRNA levels of 11 out of the 13 promoter-hypermethylated genes were restored in XL-JT and XLA-07 cells treated by 5-azacytidine (5-Aza-CR) (Figure 5C). Taken together, these results indicated that promoter hypermethylation and hypomethylation were associated with the mRNA expression levels of some genes.

### DKK2 and EN1 promoter hypermethylation, mRNA expression, and function

**Figure 6:**
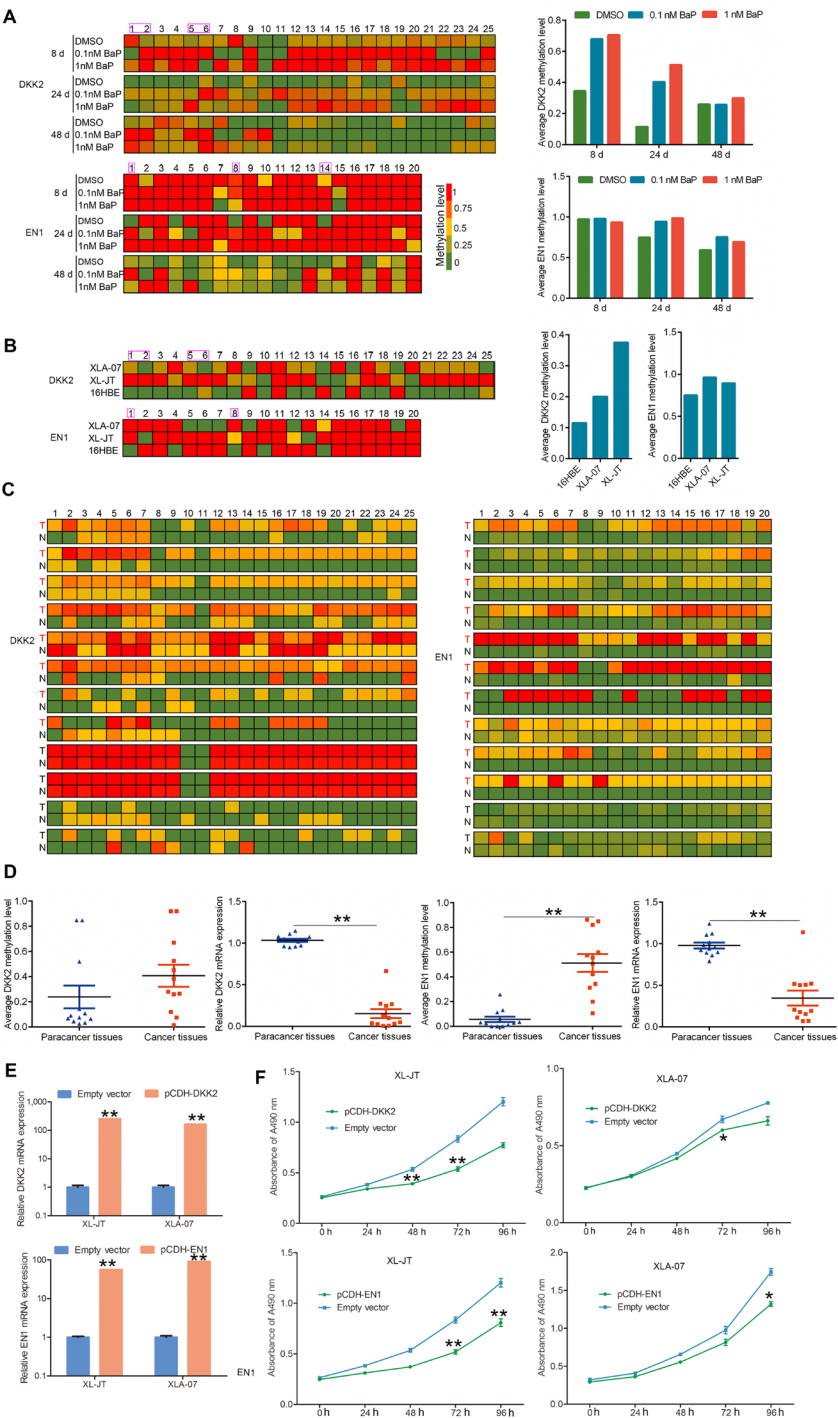
Quantitative analysis of promoter hypermethylation and mRNA expression, and functional investigation of DKK2 and EN1 **A-C.** Methylation statuses of the 25 and 20 CpG dinucleotides around the promoter region of DKK2 and EN1 were measured by BSP in BaP-exposed 16HBE cells (A), XWLC cell lines (XL-JT and XLA-07) and 16HBE cells (B), and the 12 paired XWLC tissues (C). In A and B, left: the methylation level of each CpG dinucleotide; right: the average methylation levels of total CpG dinucleotides analyzed at gene promoters. In C, methylation level of each CpG dinucleotide; T stands for XWLC tissues, N stands for adjacent normal tissues. **D.** Promoter methylation and mRNA expression levels were examined in the 12 paired XWLC tissues by BSP and qRT-PCR, respectively. Promoter methylation levels are represented by the average methylation levels of total CpG dinucleotides analyzed around the gene promoters. The results were analyzed using Student's t-test (**P < 0.01, *P < 0.05) and were expressed as the mean ± standard error (SE). **E, F.** Induced expression of DKK2 and EN1 inhibited cell proliferation in cultured XWLC cells after gene transfection. The specific over-expression of DKK2 and EN1 mRNA was confirmed by qRT-PCR after gene transfection compared to empty vector transfection (E). Cell proliferation was measured by MTS assay, and absorbance was measured at 490 nm (F). In E and F, results are presented as the mean ± standard error (SE) of triplicate experiments, which were analyzed using Student's t-test (**P < 0.01, *P < 0.05).

To quantitatively analyze the relationships between promoter hypermethylation and mRNA expression, we examined the methylation statuses of the 25 CpG dinucleotides at the DKK2 promoter and of the 20 CpG dinucleotides at the EN1 promoter by bisulfite sequencing polymerase chain reaction (BSP), respectively. The first, second, fifth, and sixth CpG dinucleotides at the DKK2 promoter as well as the first, eighth, and fourteenth CpG dinucleotides at the EN1 promoter were hypermethylated in BaP-exposed 16HBE cells, XWLC cell lines, and XWLC tissues compared with controls (Figure 6A-6B). An increase in methylation levels was observed in BaP-exposed 16HBE cells compared to the control cells. Moreover, the numbers of hypermethylated CpG dinucleotides at the DKK2 and EN1 promoters were greater in XWLC cell lines and XWLC tissues than in 16HBE cells and paracancerous tissues, respectively (Figure 6C).

In contrast, DKK2 and EN1 mRNA expression levels were decreased in BaP-exposed 16HBE cells (Figure 5C). Additionally, DKK2 and EN1 mRNA levels were significantly lower in XWLC tissues than in paracancerous tissues (Figure 6D). Taken together, these data suggested that BaP-induced hypermethylation of the DKK2 and EN1 promoters could cause gene silencing.

To investigate the functions of DKK2 and EN1, cDNAs were cloned into a pCDH-CMV-MCS-EF1-GFP-T2A-Puro lentiviral vector and transfected into XLA-07 and XL-JT cells. Flow cytometry (FCM) and qRT-PCR were used to confirm transfection efficiency and gene expression levels (Figure 6E). Cellular proliferation was significantly inhibited in XL-JT and XLA-07 cells after pCDH-DKK2 and pCDH-EN1 transfection (Figure 6F). Thus, we conjectured that the down-regulation of DKK2 and EN1 caused by promoter hypermethylation may promote cellular proliferation and might be involved in lung tumorigenesis.

### LPAR2 promoter hypomethylation and mRNA expression

**Figure 7:**
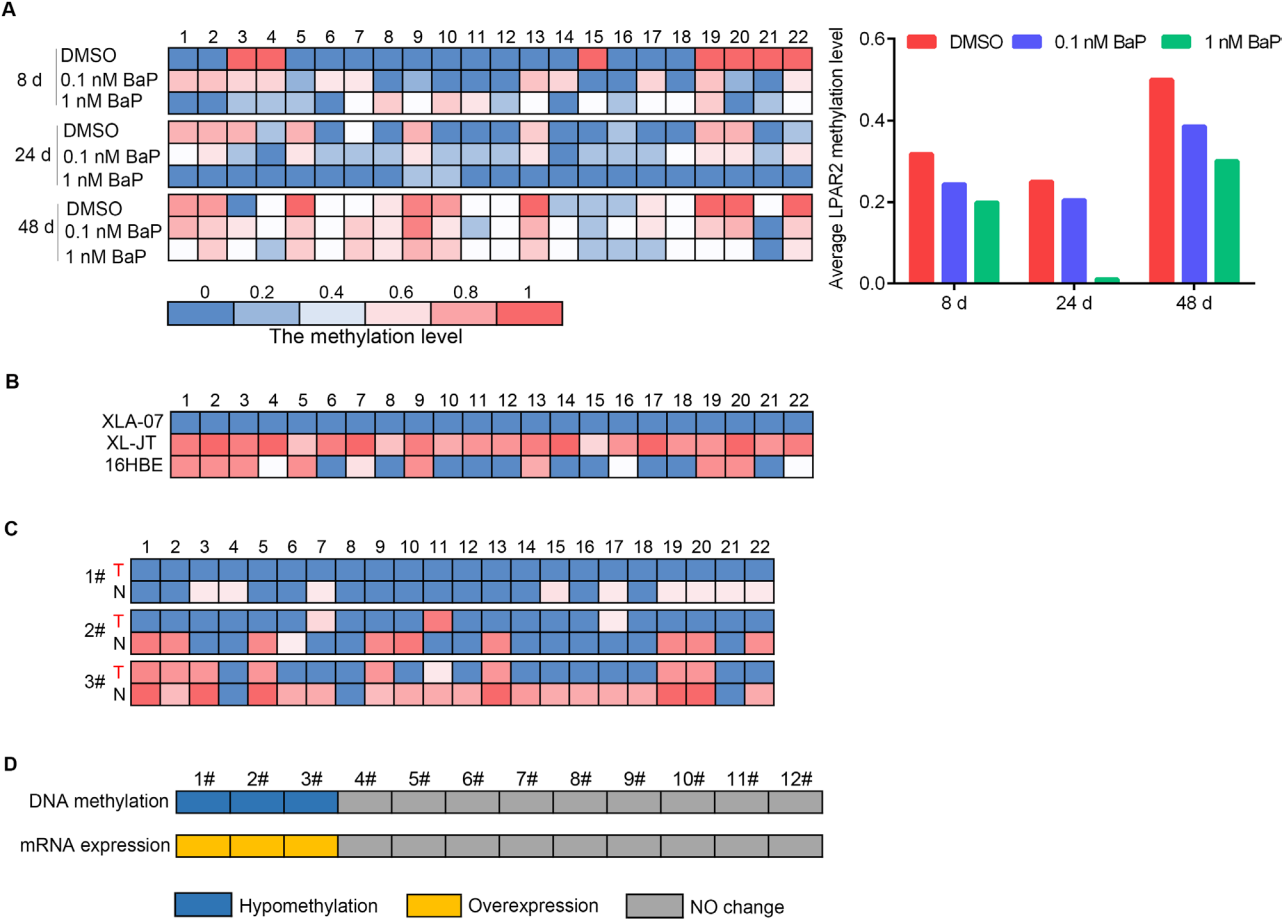
Quantitative analyses of promoter hypomethylation and mRNA expression of LPAR2 **A-C.** Methylation statuses of the 22 CpG dinucleotides around the LPAR2 promoter region were measured by BSP in BaP-exposed 16HBE cells (A), XWLC cell lines (XL-JT and XLA-07) and 16HBE cells (B), and three cases of XWLC with promoter hypomethylation (C). In A, left: the methylation level of each CpG dinucleotide; right: the average methylation levels of the 22 CpG dinucleotides analyzed around the gene promoter. In B and C, the methylation level of each CpG dinucleotide. In C, T stands for XWLC tissues, and N stands for adjacent normal lung tissues. **D.** Promoter methylation and mRNA expression levels of LPAR2 were examined in the 12 paired XWLC tissues by BSP and qRT-PCR, and the results were compared. Blue: promoter hypomethyation; yellow: mRNA over-expression; gray: no changes in promoter methylation or mRNA expression.

Promoter hypomethylation up-regulates gene expression [40], therefore, we focused on LPAR2 because its promoter was hypomethylated in BaP-exposed cells and XWLCs. The methylation status of the 22 CpG dinucleotides at the LPAR2 promoter was examined in the same samples by the same method described above. The eight CpG dinucleotides at the LPAR2 promoter were hypomethylated in BaP-exposed 16HBE cells, XWLC cell lines, and XWLC tissues (Figure 7A-7C). A decrease in the methylation level at the LPAR2 promoter was observed in BaP-exposed 16HBE cells. Hypomethylation of the LPAR2 promoter was associated with up-regulated mRNA expression in BaP-exposed 16HBE cells. Similarly, the number of hypomethylated CpG dinucleotides at the LPAR2 promoter in XLA-07 cells was greater than that in 16HBE cells. In addition, the methylation levels of the LPAR2 promoter were reduced in the three cases of the 12 XWLC tissues compared to paracancerous tissues, and LPAR2 mRNA expression levels were significantly higher in the three XWLC cases (Figure 7D). The BaP-induced hypomethylation of the LPAR2 promoter may up-regulate LPAR2 gene expression.

### Effects of vitamin C (VitC) and vitamin B6 (VB6) on genomic methylation alterations induced by BaP

**Figure 8:**
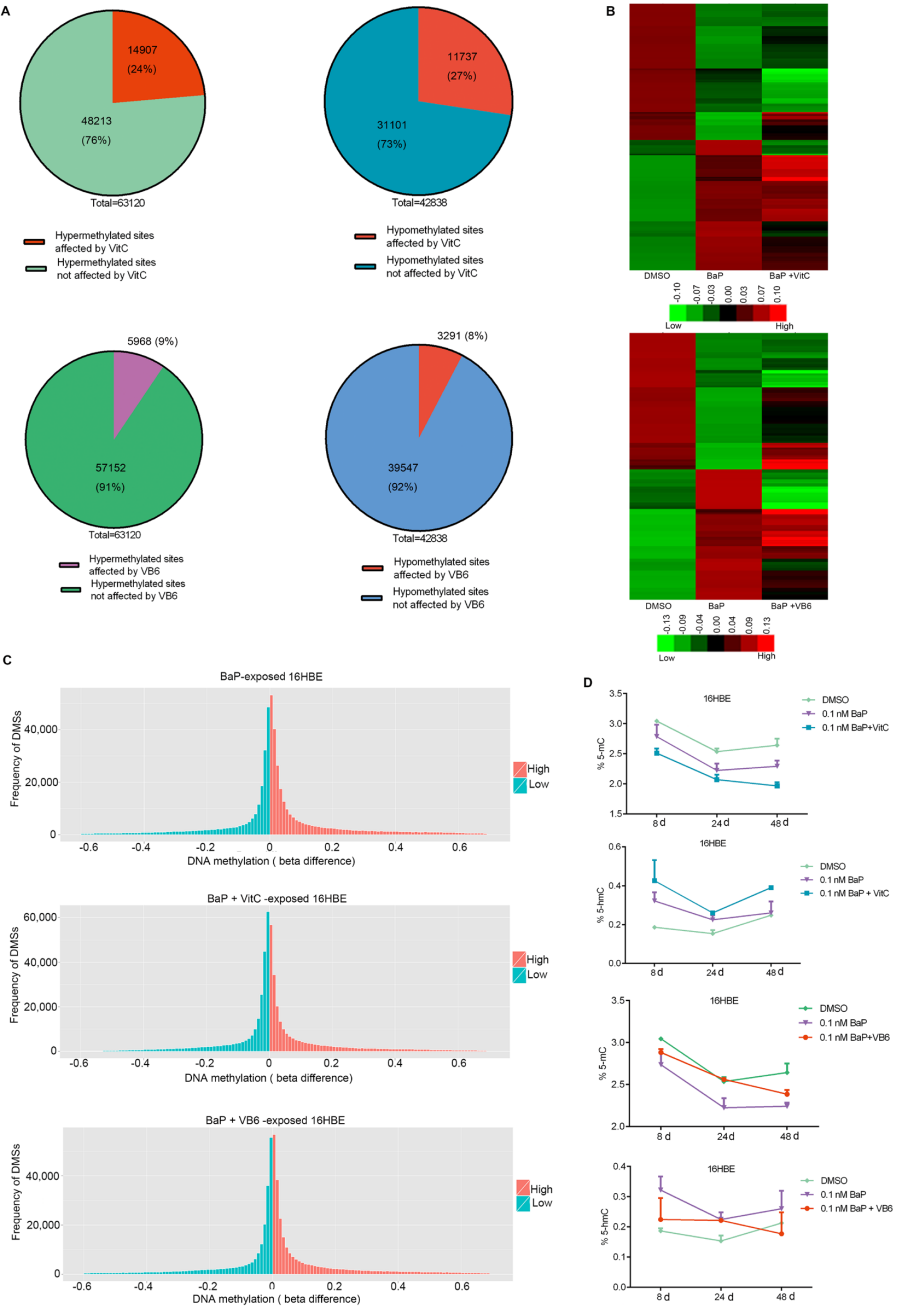
Comparison of DNA methylation among BaP-treated 16HBE cells after VitC and VB6 intervention **A.** Number of DMSs that were affected in BaP-treated 16HBE cells after VitC and VB6 intervention. **B.** Comparison of DNA methylation statuses using a heat-map. Top: treated with DMSO, BaP, and BaP plus VitC (BaP + VitC); bottom: treated with DMSO, BaP, and BaP plus VB6 (BaP + VB6). The heat map provides a visualization of β values. Red: high methylation; green: low methylation. **C.** Comparison of total DNA methylation status using normalized histogram of DMSs in 16HBE cells treated with BaP, BaP + VitC, and BaP + VB6. Red: high methylation; green: low methylation. In A-C, DMSs were obtained through comparing BaP, BaP + VitC, and BaP + VB6 treatments with DMSO treatment. **D.** 5-mC and 5-hmC levels were measured by ELISA in 16HBE cells treated with BaP, BaP + VitC, and BaP + VB6; the results were analyzed using Student's t-test (**P < 0.01, *P < 0.05). DMSO: solvent control.

VitC and VB6 can influence DNA methylation [35–37] and may be used to prevent cancer [37, 41–44]. To study whether VitC and VB6 can influence DNA methylation alterations induced by BaP exposure, genomic methylation was analyzed in IHBECs treated with a combination of BaP plus VitC and VB6. A portion of the DMSs induced by BaP were restored to normal methylation statuses by the VitC and VB6 intervention (Figure 8A-8C and Supplementary Figure S6; Supplementary Table S11; Supplementary Table S12). VitC had a much higher effect than VB6. Furthermore, genomic methylation was analyzed in murine skin treated with 5 nM BaP plus VitC for 180 days. After the VitC intervention, 88% of hypermethylated regions and 76% of hypomethylated regions that were located within annotated transcription regions (gene promoter, body, and coding regions, among others) were restored to normal methylation statuses *in vivo* (Supplementary Table S13). Thus, BaP-induced DNA methylation alterations can be reduced by combination VitC and VB6 treatment.

In addition, a slight decrease in 5-mC levels and a slight increase in 5-hmC levels were observed in BaP-exposed 16HBE cells after the VitC and VB6 intervention (Figure 8D). However, the mRNA expression levels of DNMTs and TETs were not obviously affected (Supplementary Figure S7).

### Effects of VitC and VB6 on BaP-induced alterations in promoter methylation and mRNA expression

**Figure 9:**
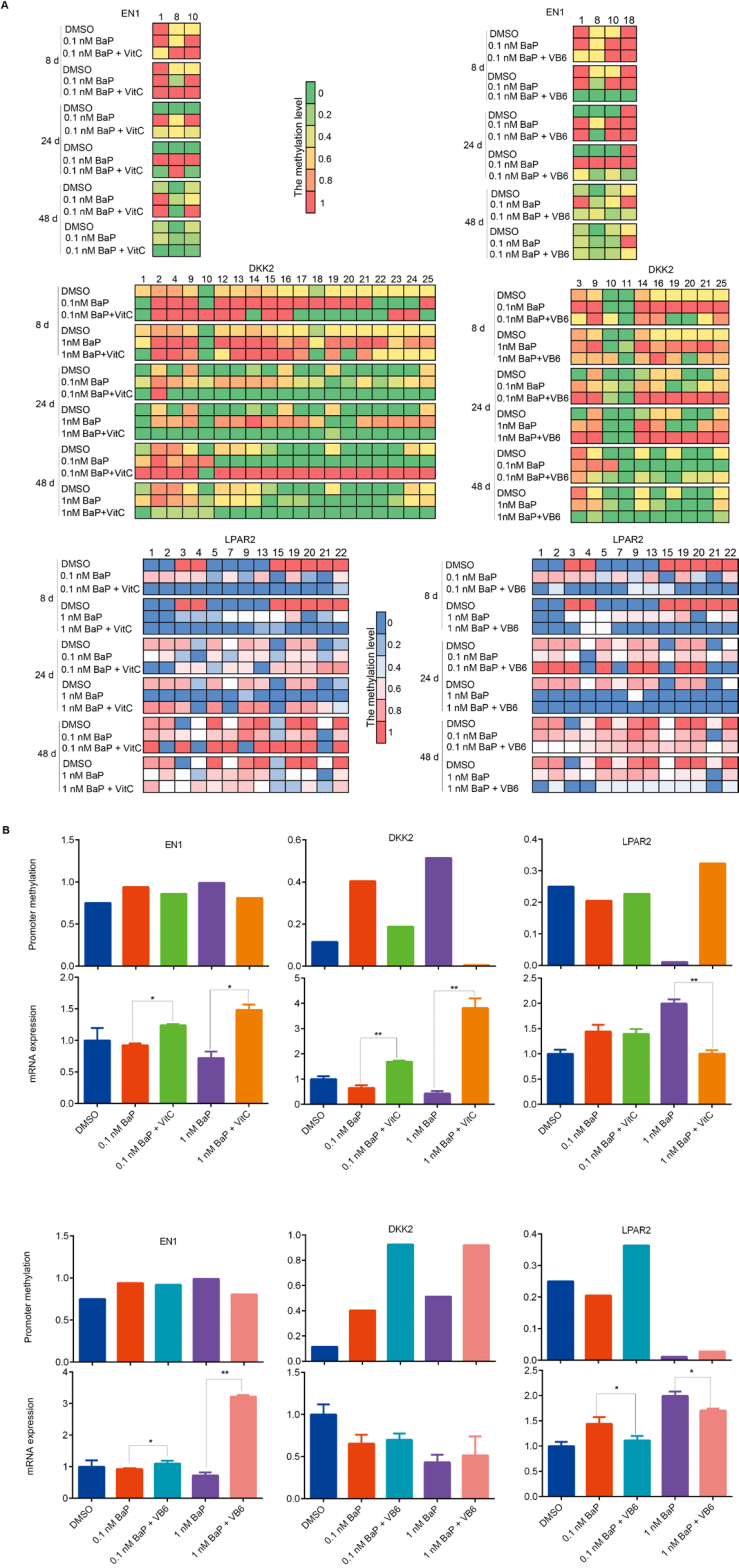
Comparison of promoter methylation and mRNA expression of EN1, DKK2, and LPAR2 after combination treatments **A.** Methylation statuses of the CpG dinucleotides around EN1, DKK2 and LPAR2 promoters were measured by BSP in 16HBE cells treated with BaP, BaP plus VitC (BaP + VitC), and BaP plus VB6 (BaP + VB6). CpG dinucleotides whose methylation statuses were changed by the VitC and VB6 intervention are shown. **B.** Integrative analyses of the promoter methylation and mRNA expression levels of EN1, DKK2, and LPAR2 in 16HBE cells treated with BaP, BaP + VitC, and BaP + VB6 for 24 days. The promoter methylation levels are presented as the average methylation levels of total CpG dinucleotides analyzed around gene promoters. The mRNA expression levels were measured by qRT-PCR.

To study whether VitC and VB6 can attenuate BaP-induced promoter methylation alterations, we measured the promoter methylation statuses of three genes (DKK2, EN1, and LPAP2) using BSP in 16HBE cells treated with BaP plus VitC or VB6. Interestingly, the methylation levels of the EN1 and DKK2 promoters were decreased by the VitC intervention, while the methylation level of the LPAR2 promoter was increased (Figure 9A). Notably, the action of VitC on DNA methylation was CpG dinucleotide specific, i.e., it specifically acts on certain sites. As expected, the mRNA expression of DKK2 and EN1 and of LPAR2 was reactivated and silenced, respectively, by the VitC intervention. However, VB6-mediated effects on DNA methylation and gene expression were complicated and far weaker than were those of VitC (Figure 9A-9B).

## DISCUSSION

In the present study, we obtained comprehensive data regarding genome-wide CpG island methylation in XWLC by microarray assay. Subsequently, we verified 17 DMRs in an expanded XWLC sample set by MSP, and the results from the microarray assay and MSP were consistent. Altered DNA methylation is an important event that plays a role in carcinogenesis. Previous studies on genomic methylation examined general lung cancers [19–22]. Our results provide new data for air pollution-related lung cancer. We found several novel tumor-specific methylated genes. The DNA methylation statuses of some genes were associated with clinicopathological characteristics of the patients. Several novel tumor-specific methylated genes showed a high positive rate in lung cancer. As an early biomarkers of cancer risk and diagnosis, DNA methylation has several advantages [26, 45, 46]. Thus, these tumor-specific methylated genes have the potential to be used as biomarkers of lung cancer in clinical application.

BaP is one of key carcinogens associated with air pollution-related lung cancer. In this study, IHBECs and mice were treated with low concentrations of BaP *in vitro* and *in vivo*, and the samples were subsequently analyzed for genomic methylation. Although BaP can directly bind to DNA, form DNA adducts, and induce gene mutations [47], the low doses of BaP used in the present study did not lead to mutations of several genes, such as TP53, KARS, and EGFR; mutations in these genes frequently occur in lung cancer (Supplementary Table S14). However, the low doses of BaP did induce DNA methylation alterations *in vitro* and *in vivo*, similar to those reported in previous studies [48–51]. Moreover, BaP exposure induced both DNA hypermethylation and hypomethylation, and DNA methylation alterations were more obvious with higher concentration of BaP and longer durations of BaP exposure. Therefore, we compared DNA methylation alterations of BaP-exposed cells with those of cultured human lung cancer cells and found that BaP- induced DNA methylation alterations occurred partially in lung cancer cells. Furthermore, lower levels of global 5-mC and higher levels of 5-hmC were observed in BaP-exposed cells, similar to those observed in XWLC cell lines and tissues. Therefore, DNA methylation alterations induced by BaP may partly explain the aberrant DNA methylation observed in XWLC.

DNA methylation and demethylation are regulated by DNMTs and TETs [18, 38, 39]. In the present study, BaP induced changes in DNMT3A, DNMT3B, and TET1 mRNA expression, a pattern also partially observed in XWLC tissues and cell lines. BaP-induced DNA methylation alterations may result from changes in DNMT and TET expression levels. However, the mechanism by which BaP induces these changes remains unclear thus far. Additionally, multiple alterations in DNA methylation, *e.g.*, global DNA hypomethylation and gene-specific hyper- and hypomethylation, appeared simultaneously in BaP-exposed cells. On one hand, BaP induced DNA hypermethylation through up-regulating certain types of DNMTs (*e.g.*, DNMT1). On the other hand, BaP could also induce DNA hypomethylation by down-regulating other types of DNMTs (*e.g.*, DNMT3A, DNMT3B) and up-regulating TETs (*e.g.*, TET1). Thus, changes in DNMTs and TETs expression alone cannot explain all of the above-described phenomena. The underlying mechanisms of BaP-induced DNA methylation alterations have not been completely elucidated. In particular, the mechanism(s) by which BaP causes gene-specific hyper- and hypomethylation and controls the targeting of specific genes remains largely unknown.

In the present study, BaP exposure induced multiple alterations in DNA methylation and DNMT and TET mRNA expression. Furthermore, these alterations occurred partially in lung cancer cells. Thus, we hypothesized that the action of BaP represents one cause of DNA methylation alterations in air pollution-related lung cancer. Not all of these alterations have functional significances or play a causative role in carcinogenesis; rather, most of these methylation alterations are only consequential events and should be considered “passenger events” in carcinogenesis. However, global hypomethylation correlates with genomic instability [17], and abnormal DNA methylation may increase the susceptibility of gene mutations caused by carcinogens [40, 52]. Intermediate DNA methylation is a conserved signature of genome regulation [53], and promoter hyper- and hypomethylation can regulate gene expressions [16, 18, 40]. Thus, we aimed to evaluate the significances of gene-specific methylation induced by BaP.

Meta-analysis of DNA methylation and mRNA expression revealed that the mRNA expression level of some genes was associated with DNA methylation statuses, particularly with promoter methylation levels. Subsequently, we found that 11 of the 13 promoter-hypermethylated genes exhibited mRNA down-regulation in BaP-exposed cells; and the mRNA expression levels of these 11 genes were increased in XWLC cell lines after demethylation by 5-Aza-CR. Moreover, two of the promoter-hypermethylated genes (DKK2 and EN1) were further studied. DKK2, a putative Wnt signaling inhibitor, is generally down-regulated in human cancers [54, 55]. EN1, a homeobox transcription factor, plays a major role in development [56]. EN1 is hypermethylated in neoplasia [57], this alteration has been identified as a diagnostic marker in colorectal cancer [58]. In this study, quantitative analyses of DNA methylation and mRNA expression confirmed that 1) promoter hypermethylation of DKK2 and EN1 could be induced by BaP, and hypermethylation also occurred in XWLC; 2) promoter hypermethylation of DKK2 and EN1 was associated with mRNA down-regulation. Additionally, DKK2 and EN1 up-regulation inhibited cell proliferation in lung cancer cells. Therefore, BaP-induced hypermethylation of the promoters of some genes may affect gene expression and cell behavior. Interestingly, the down-regulation of mRNA expression of the 11 genes was recovered in cultured lung cancer cells after 5-Aza-CR treatment. DNA methylation has been identified as a therapeutic target of epigenetic drugs for cancer therapy [59, 60]. Two drugs, 5-Aza-2'-deoxycytidine (5-Aza-CdR) and 5-Aza-CR, have been approved by the Food and Drug Administration of the United States for clinical applications, so 5-Aza-CdR and 5-Aza-CR may be considered for having possible therapeutic applications for the treatment of lung cancer.

Apart from promoter hypermethylation, BaP exposure can also induce promoter hypomethylation. Genomic analyses revealed that the LPAR2 and MAGEA1 promoters were hypomethylated in BaP-exposed cells and XWLCs; the mRNA expression of the two promoter-hypomethylated genes appeared to be up-regulated in BaP-exposed cells. LPAR2 is a member of family I of the G protein-coupled receptors and is capable of enhancing the migration, invasion and metastatic potency of cancer cells [61, 62]. Quantitative analyses confirmed that BaP-induced hypomethylation of the LPAR2 promoter was associated with mRNA up-regulation.

In this comprehensive study, BaP-induced hypermethylation and hypomethylation of the promoters of some genes are associated with gene silencing and activation, respectively, and the altered expression levels of these genes can affect the biological behavior of cells. Thus, we proposed that some DNA methylation alterations induced by BaP may serve as “drivers” and contribute to the development and progression of lung cancer.

Air pollution-related lung cancer is increasing worldwide, particularly in developing countries. Cancer prevention is extremely valuable, and some chemical agents have been identified or suggested as efficacious preventive drugs for cancer prevention. VitC and VB6 can affect DNA methylation [35–37], vitamins may provide significant protection against aberrant DNA methylation [42]. In our study, the induction of DNA methylation alterations, particularly gene-specific hyper- and hypomethylation, by BaP could be partially abated by VitC and VB6. VitC showed a stronger inhibitory action than VB6, and VitC had the same effect *in vivo*. However, the mRNA levels of DNMTs and TETs were not obviously affected by VitC and VB6. VitC promotes widespread and specific DNA demethylation in stem cells through modulating TET1 function [35, 36], and VB6 is involved in DNA methylation through participating in one-carbon metabolic pathways [37]. Therefore, VitC and VB6 may influence DNA methylation through different mechanisms, *e.g.*, modulation of TET function by VitC and regulation of enzyme substrates by VB6. It is known that VitC can protect cells from oxidative DNA damage and quench free radicals [63]. Our findings expand our knowledge of VitC and VB6 functions. Dietary VitC protects against lung cancer risk [44, 64], and VitC can selectively kill cancer cells [65]. Additionally, previous studies found a substantial decrease in the risk of lung cancer with increasing levels of VB6 [41, 43]. Therefore, we suggest that VitC and VB6 may be used as chemopreventive agents for populations that have a high risk of developing air pollution-related lung cancer, including those individuals that are very susceptible, workers exposed to high levels of air pollution, and residents living in regions with high levels of air-pollution.

Finally, we reviewed abnormally methylated genes that were related to PAHs exposure, air pollution, smoking, and lung cancer in the literature and compared those studies with our results. The 43 abnormally methylated genes identified from the literature also appeared in our data of genomic methylation profiles using BaP-exposed cells and XWLC (Supplementary Figure S8). BaP is not only a key carcinogen in polluted air but also an important carcinogen that is present in cigarette smoke. The DNA methylation alterations induced by BaP exposure may also contribute to the development and progression of lung cancer in smokers. Moreover, VitC intake is closely relates to lung cancer risk in smokers [64]. Thus, VitC and VB6 may also be used as chemopreventive drugs against lung cancer for smokers.

## MATERIALS AND METHODS

### Patient tissue samples

Lung cancer and adjacent normal lung tissues were obtained from lung cancer patients with previously untreated NSCLCs. Through rigorous screening, 14 cases of XWLC that fulfilled the criteria of genomic analysis were selected for genomic DNA methylation analysis (Supplementary Table S1A). The criteria of genomic analysis are described below: (1) residents of Xuanwei/Fuyan where there is serious air pollution with high BaP levels; (2) resided in their communities and never stayed in other regions for a long time (6 months or more); (3) previously untreated primary lung cancer. The diagnosis of lung cancer was confirmed by at least 3 pathologists; (4) the tissue samples were taken at the time of surgery and quickly frozen in liquid nitrogen. The tumor samples contained a tumor cellularity of greater than 80% and the matched control samples had no tumor content. Similarly, 51 cases of fresh tumor tissue specimen (tumor cellularity > 60%) and their matched adjacent normal counterpart were selected for other analyses (Supplementary Table S1A). The quality standard of all tissue samples were based on the guidelines of International Cancer Genome Consortium (www.ICGC.ORG/POLICIES). All of these samples were diagnosed in accordance with World Health Organization's classification and staged based on the International Union Against Cancer [66, 67]. The study was approved by the Ethics Committee for Human Medicine Research, Kunming Institute of Zoology, Chinese Academy of Sciences (Permit Number: SYDW-2012010).

Genomic DNA was extracted from all tissues by QIAamp DNA Mini Kit (Qiagen, Hilden, Germany) according to the manufacturer's instructions. The purified DNA was then quantified by NanoDrop 2000 spectrophotometer (Thermo scientific, Waltham, MA, USA), and its integrity was assessed using gel electrophoresis.

### Cell culture

Four lung cancer cell lines and two IHBEC lines were used in the present study (Supplementary Table S1B). The two XWLC cell lines, XLA-07 and XL-JT, were identified by using STR typing [68].

For carcinogen treatment, IHBECs were plated and exposed to BaP (Sigma, St. Louis, MO, USA), BaP plus VitC (ascorbic acid, Sigma), or BaP plus VB6 (pyridoxal phosphate, Sigma) for 8, 24 and 48 days, respectively. BaP was dissolved in DMSO at 0.1 nM and 1 nM concentrations. The concentrations of VitC and VB6 were 50 ng/μl and 0.05 μM, respectively. DMSO was used as the negative control. Fresh culture medium was re-supplied every two days during treatment.

For 5-Aza-CR treatment, XLA-07 and XL-JT cells were seeded and treated with 10 μM (5-Aza-CR, Sigma) for 4 days. Culture medium containing 5-Aza-CR was re-supplied every 24 hours during the treatment. The control cells were handled in the same way without 5-Aza-CR.

### Animal experiment

Six BALB/c mice were raised under standard conditions. The mice were divided into two groups (three mice in each group). To simulate the direct contact of human bronchial epithelial cells with environmental BaP, the skin of the mice was frequently treated (three times each week) with a low concentration of BaP for a long period (six months) similar as previous studies [69, 70]. In the first group, one side of the skin on the back of each mouse was exposed to 5 nM BaP dissolved in acetone three times each week for 180 days; another side of the skin on the back of the same mice was treated with acetone as the solvent control; The abdominal skin of the same mice were treated with 5 nM BaP plus 50 ng/μl VitC. In the second group, mice were treated with 50 nM BaP and acetone in a similar fashion as the first group. Genomic DNA was extracted from micro-dissected skin tissues. Samples from the same mice were paired for comparative analysis. Samples from the first group were used for genomic methylation analysis; samples from the first and second group underwent a gene mutation test. The animal experiments were carried out in strict accordance with institutional guidelines and were approved by the Ethics Committee for Animal Experimentation, Kunming Institute of Zoology (Permit Number: SYDW-2012010).

### Genomic methylation profiling

Genomic methylation analysis of the 14 XWLC and paracancerous tissues was carried out using a Roche NimbleGen Human DNA Methylation 3×720K CpG Island Plus RefSeq Promoter Array Chip (Roche, Basel, Switzerland) at CapitalBio Technology (Beijing, China; http://www.capitalbio.com). The 3×720K microarrays focused on biologically significant genomic regions, including 27,728 annotated CpG islands and 22,532 RefSeq gene promoters (UCSC, hg18), for unbiased discovery of methylated DNA regions. DNA methylated peaks were identified using the following parameters: sliding window of 750 bp, P-value minimum cut-off (−log10) of 2.0 (peak score ≥ 2), and a minimum of five features per peak.

DNA methylation profiling of cultured cells was performed using an Illumina Infinium® Human Methylation 450 BeadChip Array (Illumina Inc., San Diego, CA, USA) at Shanghai Biotechnology Corporation (Shanghai, China; http://www.shanghaibiotech.com) according to the manufacturer's specifications (Illumina). DMSs were identified using the following parameters: |beta difference| > 0.1 compared to IHBECs treated by BaP for 8 days; |beta difference| > 0.2 compared to IHBECs treated with BaP for 24 days and compared to lung cancer cell lines.

MeDIP-Seq was performed to analyze genome-wide methylation of the mouse samples at Shanghai Biotechnology Corporation using a MagMeDIP Kit (Diagenode, Denville, NJ, USA) according to the manufacturer's specifications. Statistically significant peaks at a P-value < 5% were identified using the Cummerbund package in R [71]. Peaks were matched with adjacent annotated genes.

The differentially methylated genes were subjected to GO and KEGG pathway analyses using Mas 3.0 Molecule Annotation System software (http://bioinfo.capitalbio.com/mas3).

### Methylation analysis of specific sites

For bisulfite modification, genomic DNA was treated with 3 M sodium bisulfite for 16 hours at 56°C to convert unmethylated cytosine in the genomic DNA to uracils, while methylated cytosine were not converted. A Wizard DNA Clean-Up System Kit (Promega, Madison, WI, USA) was used to purify the bisulfite-modified DNA. The bisulfite-treated DNA was use for MSP and BSP.

For MSP, primers were designed using Methyl Primer Express Software v1.0 (Applied Biosystems, Foster City, CA, USA). MSP was performed in 25 μl reaction volumes. The primer information is provided in Supplementary Table S15. The PCR products were subsequently checked using 2.0% agarose gel electrophoresis. A water blank was used as a negative control.

For BSP, the detailed methylation status of CpG sites was characterized in the candidate fragments through bisulfite clone sequencing. The CpG-free universal primers are listed in Supplementary Table S15. Bisulfite-treated DNA was amplified using touchdown PCR. The PCR products were cloned using pMD^TM^18-T Vector Cloning Kit (TaKaRa, Tokyo, Japan) according to the manufacturer's protocol and transformed into DH5a competent cells (Tiangen, Beijing, China). Eight clones were chosen for DNA sequencing in each sample. Sequence analyses and quality assessments were performed using BiQ Analyzer software [72]. The methylation level of each CpG dinucleotide was calculated as the ratio of positive clone (methylated CpG dinucleotide) numbers to eight (examined clone numbers). The average methylation level of total CpG dinucleotides was calculated as the ratio of the total number of positive clones to eight multiplied by the total number of CpG dinucleotides that were analyzed.

### Global 5-mC and 5-hmC measurement

The 5-mC DNA ELISA Kit and Quest 5-hmC™DNA ELISA Kit (Zymo Research, Orange Country, CA, USA) were respectively used to quantify levels of 5-mC and 5-hmC in the genomic DNA according to the instructions. The 5-mC and 5-hmC amounts were measured in proportion to the optical density (OD) intensity at 450 nm with a microplate reader (model 680, Bio-Rad Laboratories, Berkeley, CA, USA). Each sample was measured in duplicate.

### MRNA expression profiling

Total RNA was isolated using the Trizol reagent (TaKaRa) following the manufacturer's protocol. Determination of mRNA profiling was performed in 16HBE, XLA-07, XL-JT and EPLC-32M1 using Agilent 60 K Human Gene Expression array by CapitalBio Corporation (Beijing, China; http://www.capitalbio.com).

### QRT-PCR

The cDNA was synthesized by M-MLV Reverse Transcriptase (Promega) using random primer. QRT-PCR was carried out in triplicate for the target genes using FastStart Universal SYBR Green Master (Roche) on the StepOne Real time PCR System (Applied Biosystems). All primers were listed in Supplementary Table S15. GAPDH was used as the reference internal control. Fold change of gene expression was calculated with the 2^−ΔΔCT^ method.

### Vector construction and cell transduction

Full-length human DKK2 and EN1 genes were obtained via PCR from 16HBE cDNA. The PCR primers containing engineered restriction enzyme sites were shown in Supplementary Table S15. pMD^TM^18-T Vector (TaKaRa) was used as an intermediate vector. Full length DKK2 and EN1 cDNAs were then subcloned into pCDH-CMV-MCS-EF1-GFP-T2A-Puro lentiviral vector (System Biosciences, Whisman, CA, USA), respectively. Subsequently, virus packaging and infection were performed according to manufacturer's protocol. All constructs were verified by DNA sequencing. Lentiviral constructs were introduced into 293TN cells with psPAX and pMD2.G vectors using ViaFect™ Transfection Reagent (Promega) per manufacturer's protocol, and culture media were harvested and filtered through a 0.45 μm filter. The cultured lung cancer cells were infected with packaged lentivirus. After 72 hours transduction, green fluorescent protein expression was examined by FCM analysis and mRNA expressions of target genes were confirmed via qRT-PCR.

### Cell proliferation assay

Transduced cells were seeded in 96-well plates at a density of 1×10^3^ cells per well and a MTS (3-(4,5-dimethylthiazol-2-yl)-5-(3-carboxymethoxyphenyl)-2-(4-sulfo- phenyl)-2H-tetrazolium, inner salt, Promega) assay was performed every 24 hours. Proliferating cells were measured by absorbance at 490 nm using a microplate reader (model 680, Bio-Rad Laboratories).

### Statistical analysis

The data were analyzed using the SPSS (Statistical Package for the Social Sciences) 17.0 software package (Chicago, IL, USA). The statistical analyses of relation between patients' clinicopathologic characteristics and methylation statuses were analyzed using Pearson Chi-square or Fisher's exact probability test. A value of P < 0.05 was considered significant. The measurement data (5-mC and 5-hmC levels, mRNA levels, quantitative results of DNA methylation, and MTS assay results) were analyzed by Student's t-test or a one-way ANOVA.

## CONCLUSIONS

**Figure 10:**
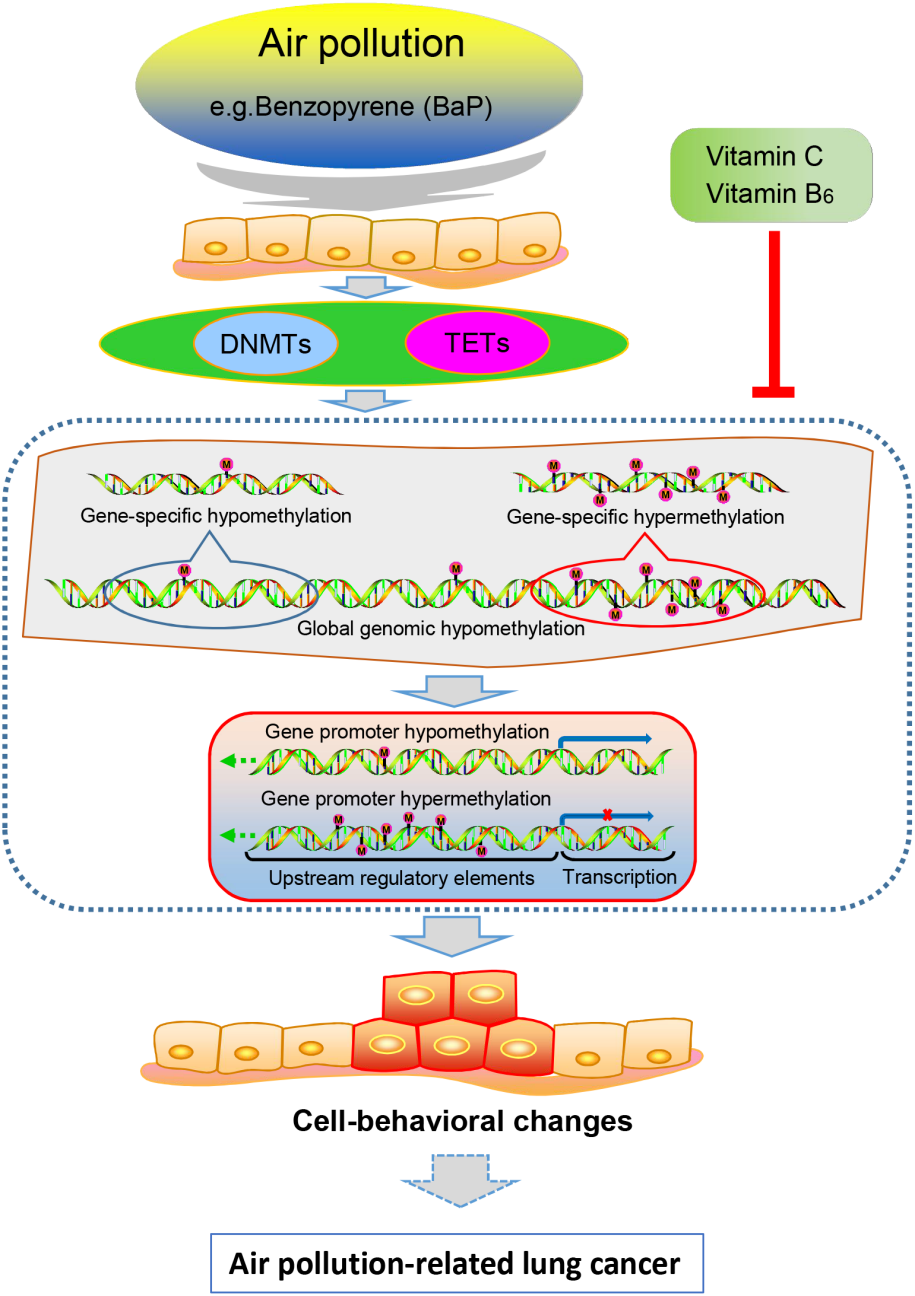
Proposed model for the relationships between air pollution-related lung cancer and BaP-induced DNA methylation alterations

We systematically and comparatively studied DNA methylation alterations in XWLC tissues, cultured XWLC cells, and BaP-treated cells and murine samples (Supplementary Figure S9). First, we collected comprehensive data regarding genome-wide CpG island methylation in air pollution-related lung cancer and found some novel tumor-specific methylated genes. Several of these novel tumor-specific methylated genes have the potential to be biomarkers of lung cancer. Second, BaP exposure induced multiple alterations in DNA methylation and in the mRNA expression level of DNMTs and TETs; these alterations partially occurred in XWLC. Third, promoter methylation alterations induced by BaP may regulate abnormal expression in some genes, and abnormal gene expressions can affect the biological behaviors of cells. Additionally, alterations in promoter methylation and mRNA expressions induced by BaP exposure could be partially restored by VitC and VB6. Thus, we hypothesized that DNA methylation alterations induced by the environmental carcinogen BaP are one of mechanisms underlying the development and progression of air pollution-related lung cancer and that the carcinogenic action of this environmental carcinogen can be reduced by VitC and VB6 (Figure 10). VitC and VB6 may be used as chemopreventive agents for air pollution-related lung cancer.

## SUPPLEMENTARY FIGURES AND TABLES




























