# Supplementary material for: Air pollution and DNA methylation alterations in lung cancer: A systematic and comparative study
Source: Oncotarget. 2016 Nov 25;8(1):1369–91. doi: 10.18632/oncotarget.13622 (PMC5352062; doi:10.18632/oncotarget.13622)
Supplement: Supplementary file 1 [file oncotarget-08-1369-s001.pdf]

Air pollution and DNA methylation alterations in lung cancer: A systematic and comparative study

SUPPLEMENTARY FIGURES AND TABLES

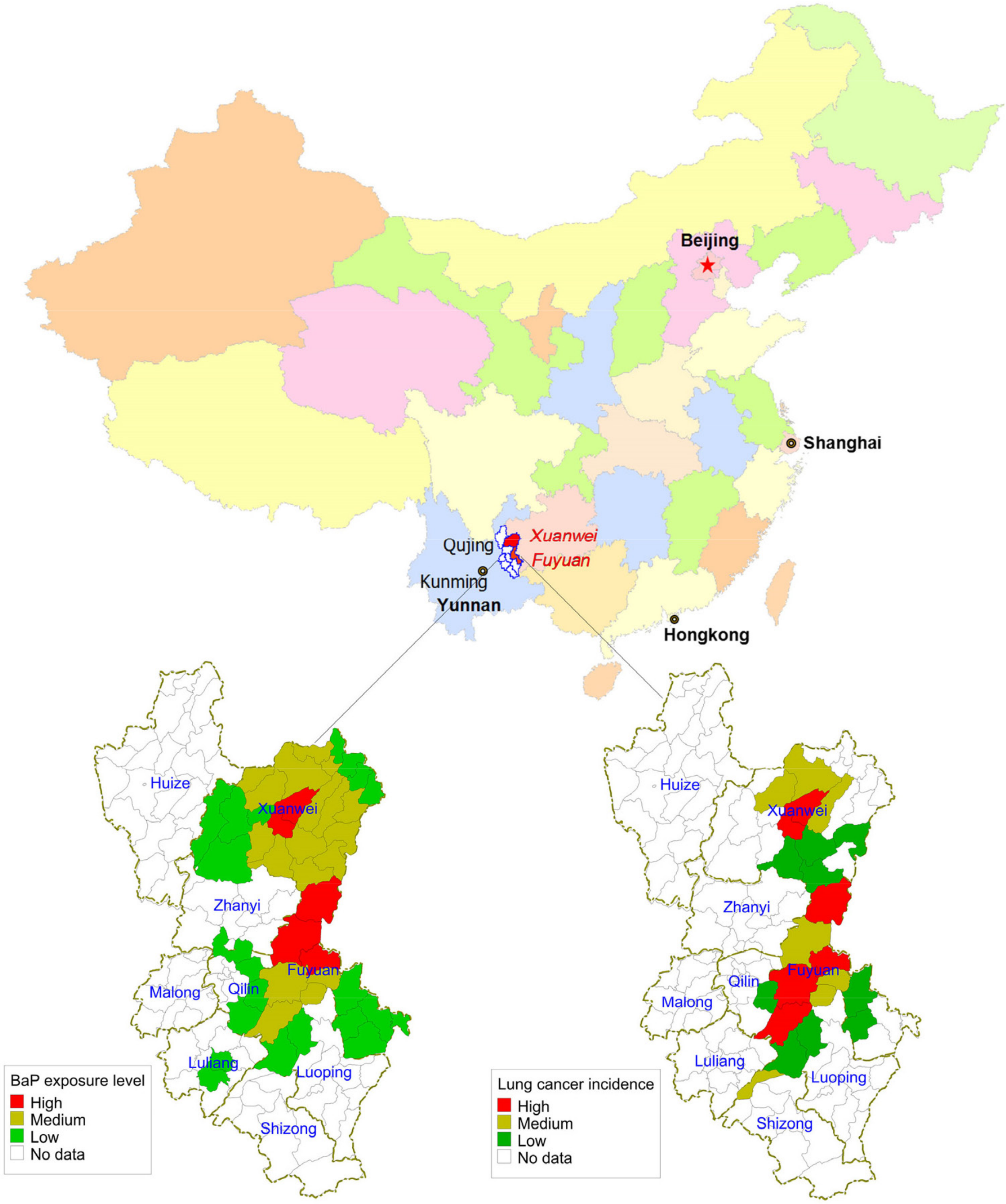

Supplementary Figure S1: Lung cancer incidence and BaP exposure levels in Xuanwei and Fuyuan. Both Xuanwei and Fuyuan are located in the northeast region of Yunnan Province, China. Xuanwei and Fuyuan are rich in smoky coal mines. The high levels of indoor and outdoor air pollution are caused by burning smoky coal. BaP exposure levels are divided into three categories: high, medium, and low. The incidence of lung cancer is strongly correlated with the BaP exposure levels.

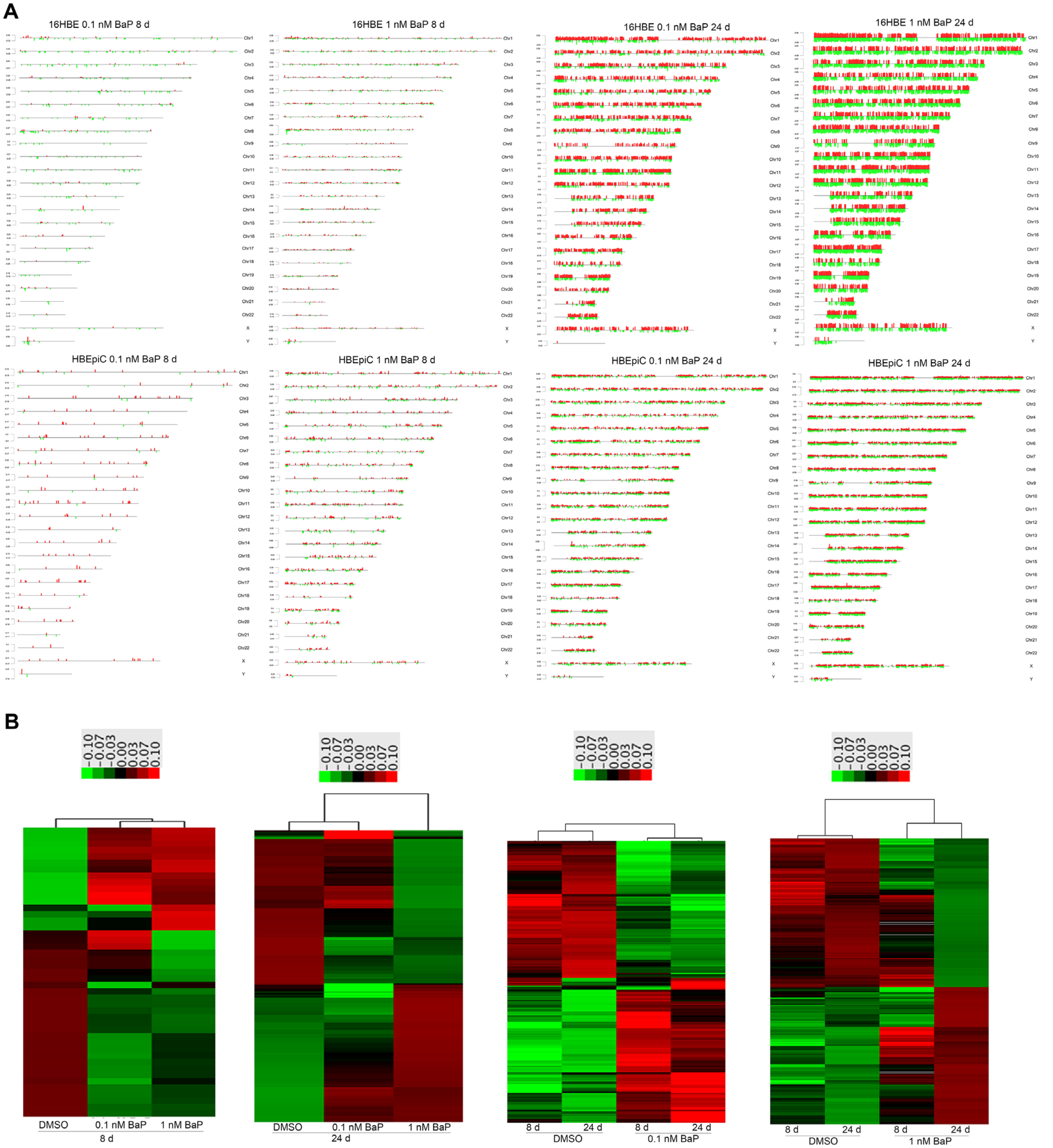

Supplementary Figure S2: Chromosome distribution of differentially methylated sites and heat map of DNA methylation in BaP-exposed cells. A. Chromosome distribution of differentially methylated sites (DMSs) in BaP-exposed 16HBE and HBEpiC cells. Red and green vertical lines represent, respectively, hypermethylated and hypomethylated sites. **B.** Heat map comparison of total DNA methylation statuses in 16HBE cells treated with BaP at varying concentrations and times. The heat map provides a visualization of β values. Red: high methylation; green: low methylation.

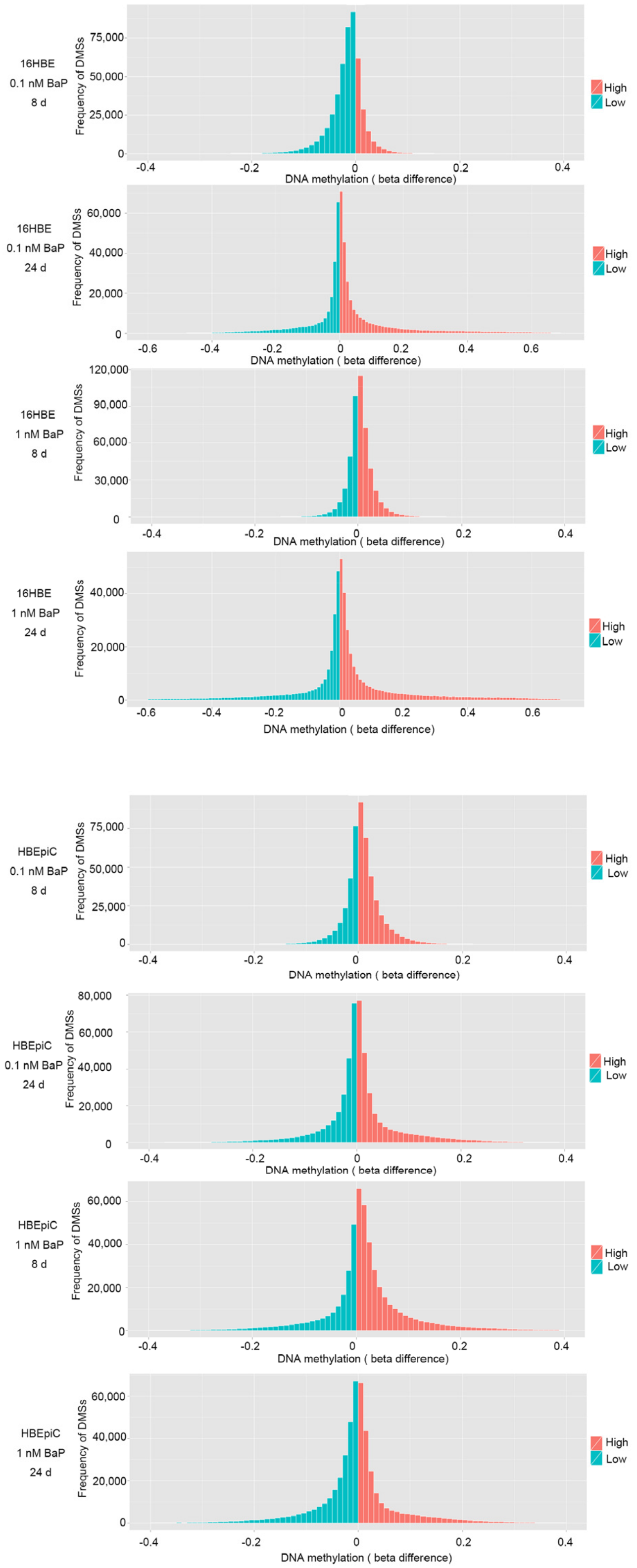

Supplementary Figure S3: Normalized histogram of differentially methylated sites in BaP-exposed IHBECs. Comparison of total DNA methylation statuses using a normalized histogram of differentially methylated sites (DMSs) in 16HBE and HBEpiC cells treated with BaP at varying concentrations and times. Red: hypermethylated CpGs; green: hypomethylated CpGs.

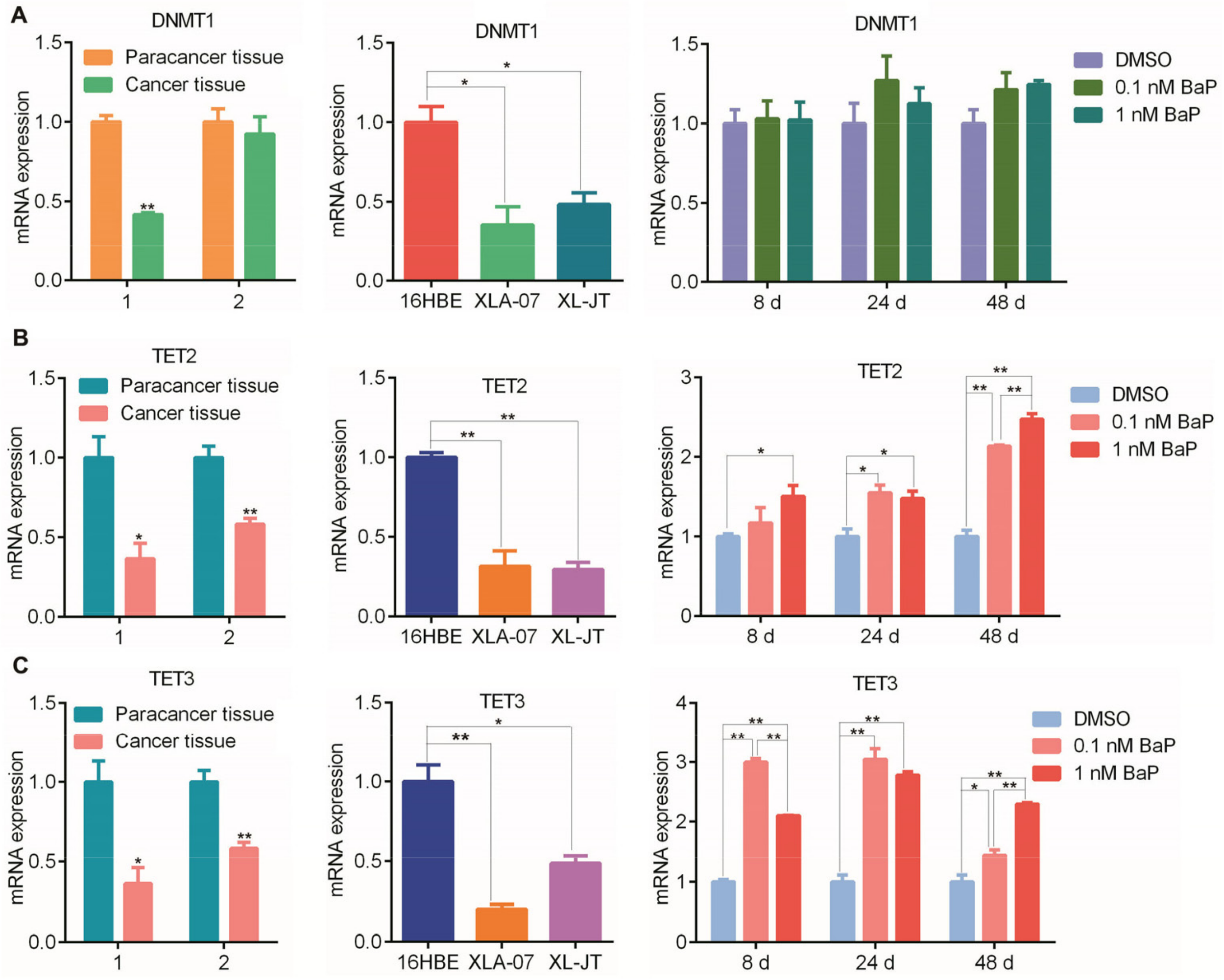

Supplementary Figure S4: Relative mRNA levels of DNMT1, TET2, and TET3. DNMT1 **A.**, TET2 **B.**, and TET3 **C.** mRNA levels were examined by qRT-PCR in paired XWLC tissues, cultured XWLC cells, and BaP-exposed 16HBE cells. For BaP-exposed 16HBE cells, DMSO was used as the control. The results were analyzed using Student's t-test (**P < 0.01, *P < 0.05).

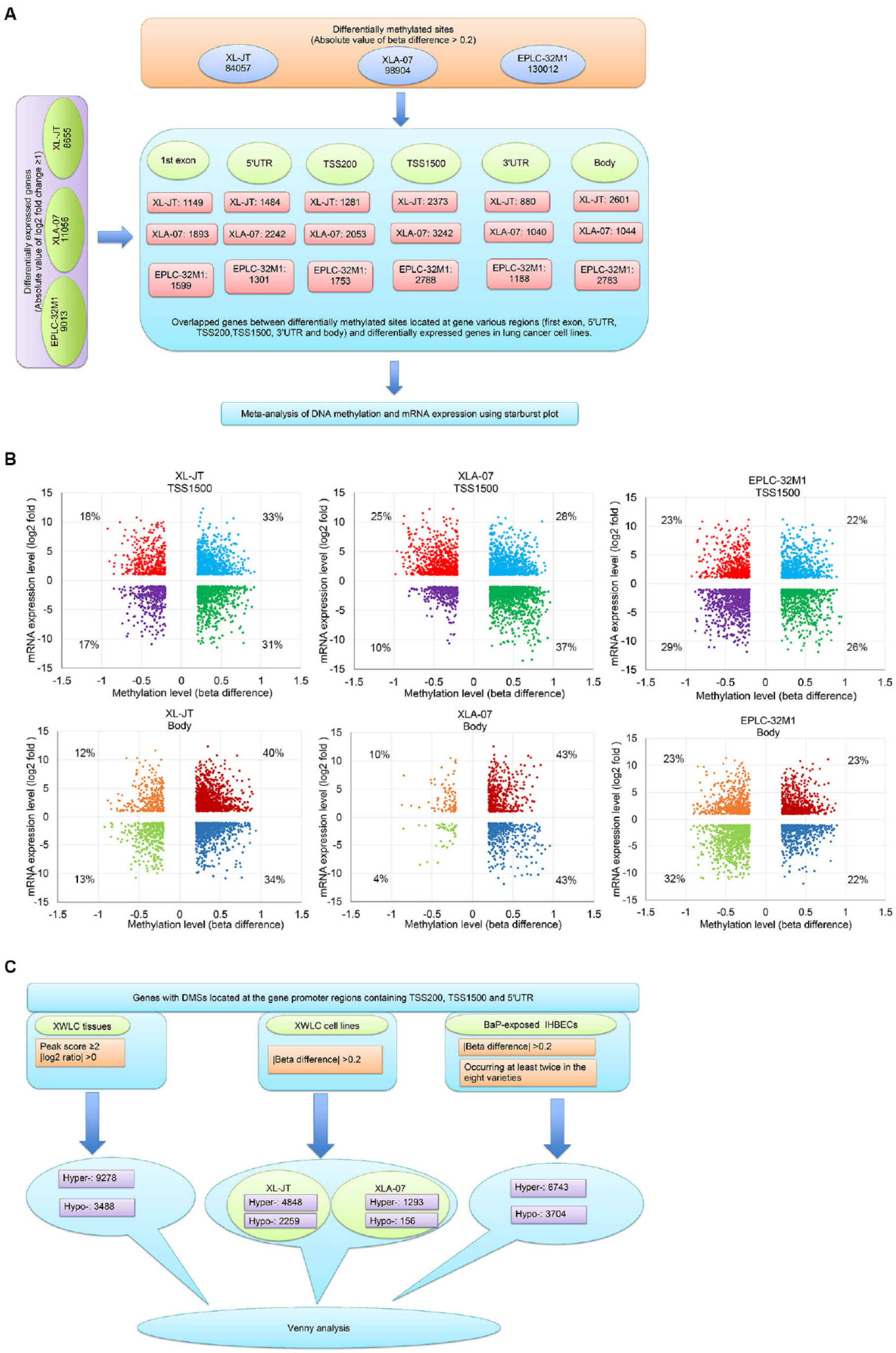

Supplementary Figure S5: Strategies of selecting genes for meta-analysis and comparative analysis, and results of meta-analysis. A. The strategy used to select genes for meta-analysis of DNA methylation and mRNA expression. **B.** Starburst plot of genes showing the relations between methylation levels (x-axis) at various regions (gene TSS1500 and body) and mRNA levels (y-axis) in lung cancer cell lines. Genes were selected according to (A). **C.** The strategy used to select genes for comparative analyses of promoter-hypermethylated and hypomethylated genes among XWLC tissues, XWLC cell lines, and BaP-exposed IHBECs.

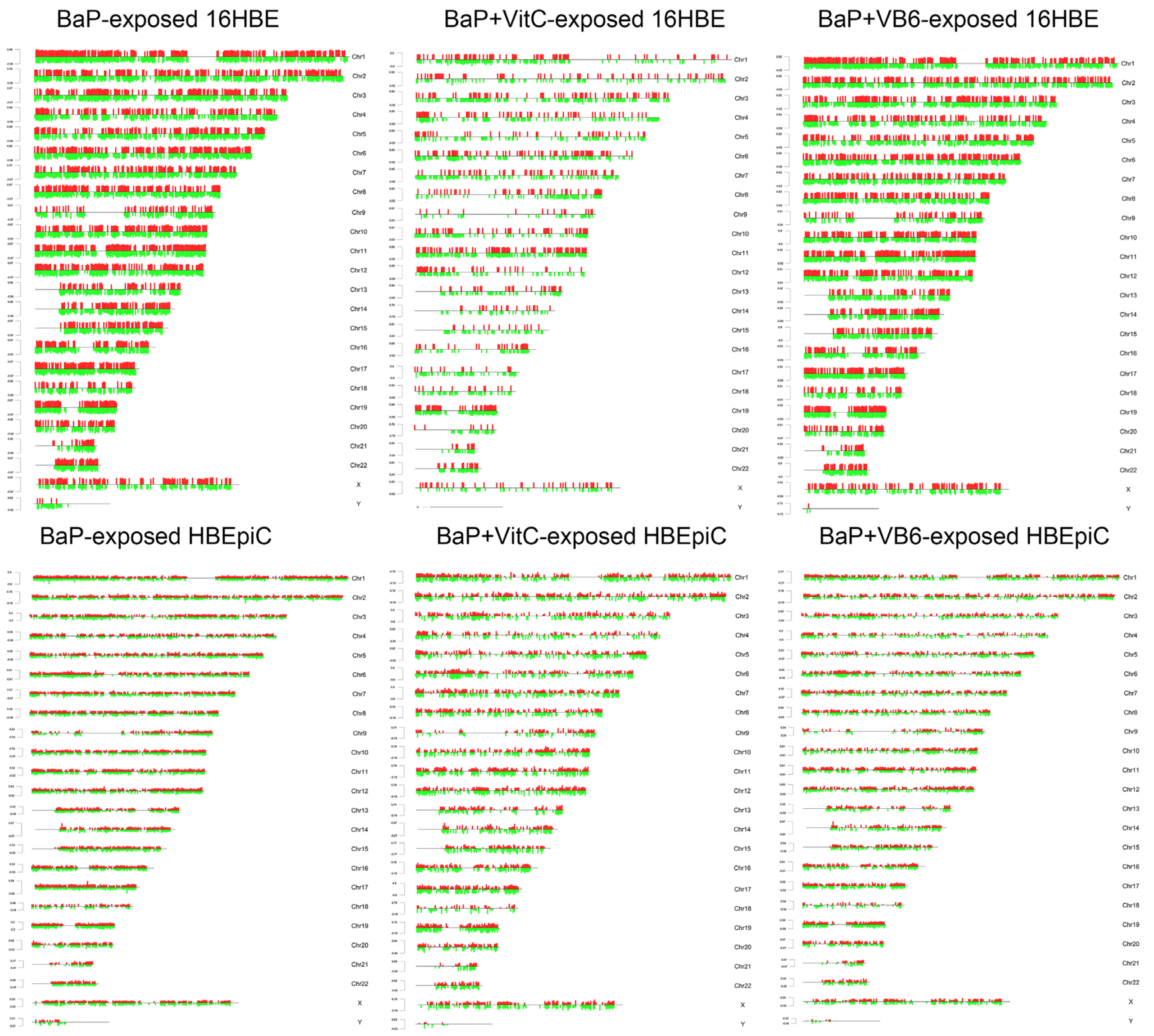

Supplementary Figure S6: Chromosome distribution of differentially methylated sites in IHBECs treated with BaP plus VitC and VB6. Red and green vertical lines represent hypermethylated and hypomethylated sites, respectively. BaP plus VitC: BaP + VitC; BaP plus VB6: BaP + VB6.

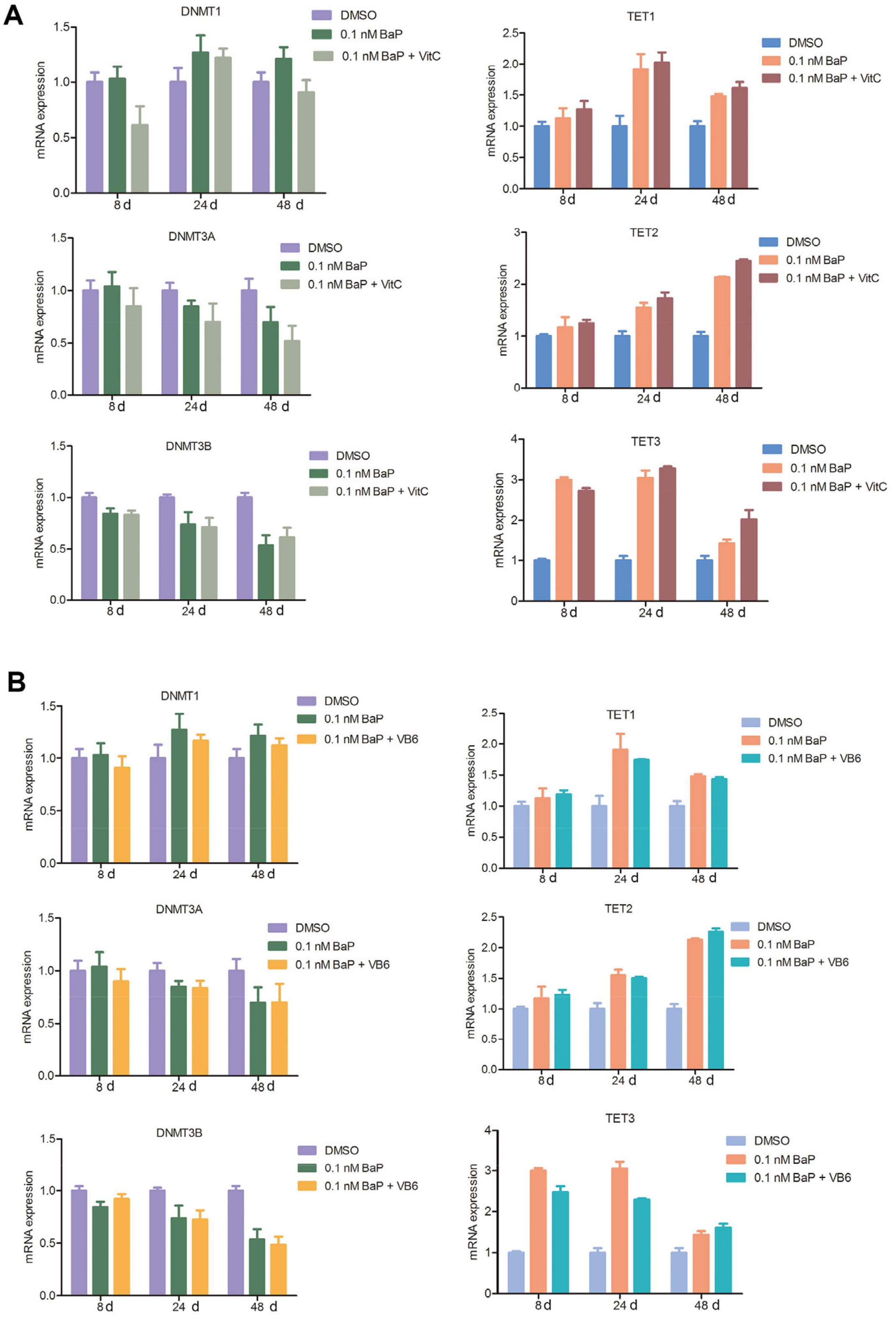

Supplementary Figure S7: Relative mRNA levels of DNMTs and TETs after BaP plus VitC and VB6 treatments. A. Comparison of DNMT1, DNMT3A, DNMT3B, TET1, TET2, and TET3 mRNA levels among 16HBE cells treated with BaP plus VitC (BaP + VitC), BaP, and DMSO. **B.** Comparison of DNMT1, DNMT3A, DNMT3B, TET1, TET2, and TET3 mRNA levels among 16HBE cells treated with BaP plus VB6 (BaP + VB6), BaP, and DMSO. In A-B, mRNA levels were measured by qRT-PCR, and the results were analyzed using Student's t-test (**P < 0.01, *P < 0.05).

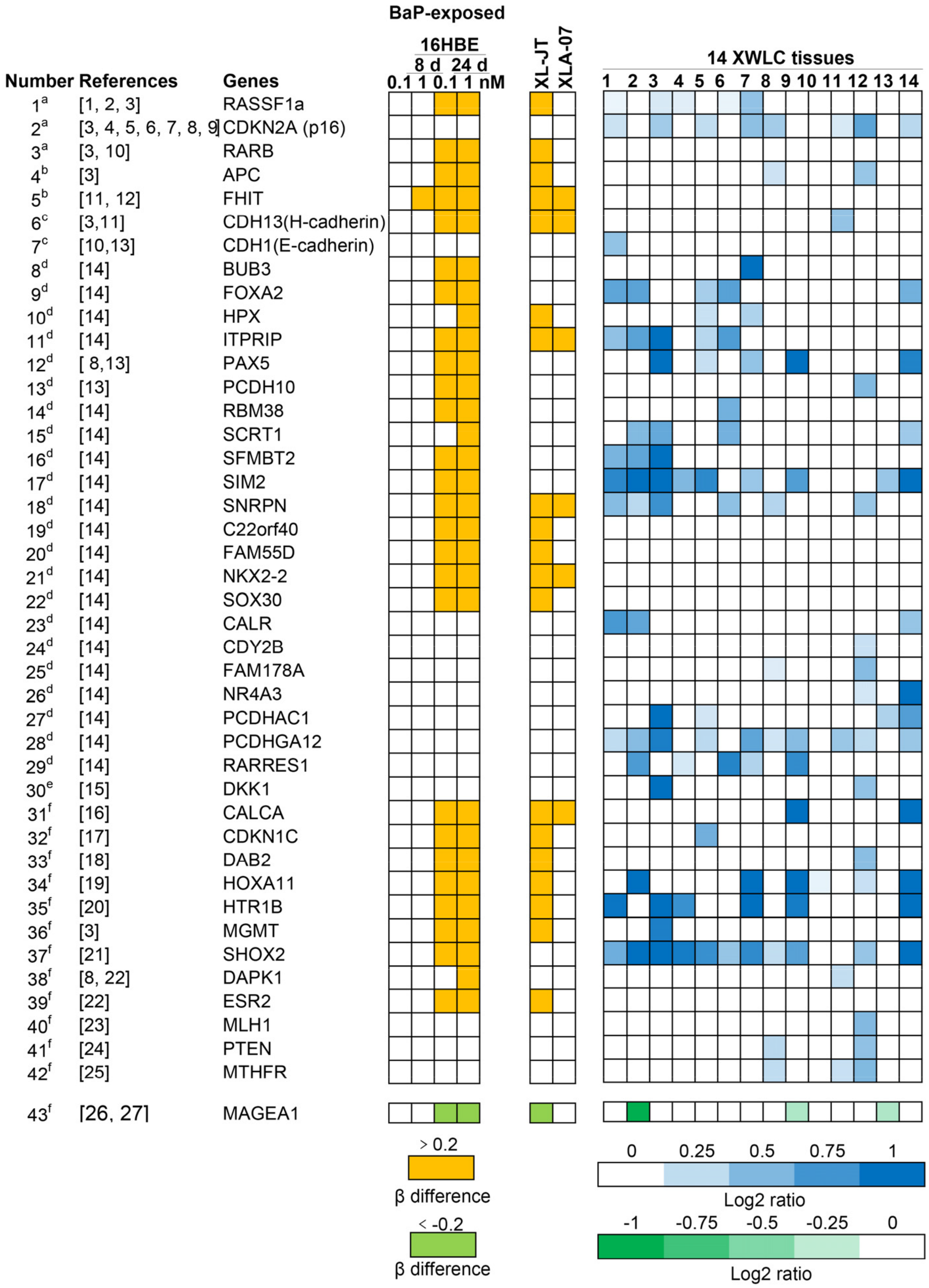

Supplementary Figure S8: Differentially methylated genes selected from the literature were compared with our methylation microarray data.

These selected genes are associated with BaP exposure, air pollution, and lung cancer.
a. Differentially methylated genes were associated with PAHs exposure, smoking and lung cancer.
b. Differentially methylated genes were associated with smoking and lung cancer.
c. Differentially methylated genes were associated with PAHs exposure and lung cancer.
d. Differentially methylated genes were associated with PAHs exposure.
e. Differentially methylated genes were associated with smoking.
f. Differentially methylated genes were associated with lung cancer.

REFERENCES

1. He Z, Duan H, Zhang B, Li M, Chen L, Zhang B, et al. CpG site-specific RASSF1a hypermethylation is associated with occupational PAH exposure and genomic instability. Toxicol. Res. The Royal Society of Chemistry; 2015;4:848–57.
2. Toyooka S, Suzuki M, Tsuda T, Toyooka KO, Maruyama R, Tsukuda K, et al. Dose effect of smoking on aberrant methylation in non-small cell lung cancers. Int. J. Cancer. 2004;110:462–4.
3. Toyooka S, Maruyama R, Toyooka KO, McLerran D, Feng Z, Fukuyama Y, et al. Smoke exposure, histologic type and geography-related differences in the methylation profiles of non-small cell lung cancer. Int. J. Cancer. 2003;103:153–60.
4. Hanabata T, Tsukuda K, Toyooka S, Yano M, Aoe M, Nagahiro I, et al. DNA methylation of multiple genes and clinicopathological relationship of non-small cell lung cancers. Oncol Rep. 2004;12:177–80.
5. Yang P, Ma J, Zhang B, Duan H, He Z, Zeng J, et al. CpG site-specific hypermethylation of p16 INK4α in peripheral blood lymphocytes of PAH-exposed workers. Cancer Epidemiol. Biomarkers Prev. 2012;21:182–90.
6. Belinsky SA, Snow SS, Nikula KJ, Finch GL, Tellez CS, Palmisano WA. Aberrant CpG island methylation of the p16(INK4a) and estrogen receptor genes in rat lung tumors induced by particulate carcinogens. Carcinogenesis. 2002;23:335–9.
7. Belinsky SA, Nikula KJ, Palmisano WA, Michels R, Saccomanno G, Gabrielson E, et al. Aberrant methylation of p16(INK4a) is an early event in lung cancer and a potential biomarker for early diagnosis. Proc. Natl. Acad. Sci. U. S. A. 1998;95:11891–6.
8. Belinsky SA, Grimes MJ, Casas E, Stidley CA, Franklin WA, Bocklage TJ, et al. Predicting gene promoter methylation in non-small-cell lung cancer by evaluating sputum and serum. Br. J. Cancer. 2007;96:1278–83.
9. Rauch T a, Zhong X, Wu X, Wang M, Kernstine KH, Wang Z, et al. High-resolution mapping of DNA hypermethylation and hypomethylation in lung cancer. Proc. Natl. Acad. Sci. U. S. A. 2008;105:252–7.
10. White AJ, Chen J, Teitelbaum SL, McCullough LE, Xu X, Hee Cho Y, et al. Sources of polycyclic aromatic hydrocarbons are associated with gene-specific promoter methylation in women with breast cancer. Environ. Res. 2016;145:93–100.
11. De Fraipont F, Moro-Sibilot D, Michelland S, Brambilla E, Brambilla C, Favrot MC. Promoter methylation of genes in bronchial lavages: A marker for early diagnosis of primary and relapsing non-small cell lung cancer? Lung Cancer. 2005;50:199–209.
12. Kim H, Young MK, Jin SK, Lee H, Park JH, Young MS, et al. Tumor-specific methylation in bronchial lavage for the early detection of non-small-cell lung cancer. J. Clin. Oncol. 2004;22:2363–70.
13. Damiani LA, Yingling CM, Leng S, Romo PE, Nakamura J, Belinsky SA. Carcinogen-induced gene promoter hypermethylation is mediated by DNMT1 and causal for transformation of immortalized bronchial epithelial cells. Cancer Res. 2008;68:9005–14.
14. Zeng J, Zhang B, Yang P, Xiao Y, Wei Q, Wang Q, et al. [A genome-wide screen for promoter-specific sites of differential DNA methylation during human cell malignant transformation in vitro]. Zhonghua Yu Fang Yi Xue Za Zhi. 2011;45:404–9.
15. Liu F, Killian JK, Yang M, Walker RL, Hong J a, Zhang M, et al. Epigenomic alterations and gene expression profiles in respiratory epithelia exposed to cigarette smoke condensate. Oncogene. 2010;29:3650–64.
16. Ji M, Guan H, Gao C, Shi B, Hou P. Highly frequent promoter methylation and PIK3CA amplification in non-small cell lung cancer (NSCLC). BMC Cancer. 2011;11:147.
17. Kobatake T, Yano M, Toyooka S, Tsukuda K, Dote H, Kikuchi T, et al. Aberrant methylation of p57KIP2 gene in lung and breast cancers and malignant mesotheliomas. Oncol. Rep. 2004;12:1087–92.
18. Yano M, Toyooka S, Tsukuda K, Dote H, Ouchida M, Hanabata T, et al. Aberrant promoter methylation of human DAB2 interactive protein (hDAB2IP) gene in lung cancers. Int. J. Cancer. 2005;113:59–66.
19. Hwang JA, Lee BB, Kim YJ, Park SE, Heo K, Hong SH, et al. HOXA11 hypermethylation is associated with progression of non-small cell lung cancer. Oncotarget. 2013;4:2317–25.

20. Takai D, Yagi Y, Wakazono K, Ohishi N, Morita Y, Sugimura T, et al. Silencing of HTR1B and reduced expression of EDN1 in human lung cancers, revealed by methylation-sensitive representational difference analysis. Oncogene. 2001;20:7505–13.
21. Konecny M, Markus J, Waczulikova I, Dolesova L, Kozlova R, Repiska V, et al. The value of SHOX2 methylation test in peripheral blood samples used for the differential diagnosis of lung cancer and other lung disorders. Neoplasma. 2016;63:246-53.
22. Fujiwara K, Fujimoto N, Tabata M, Nishii K, Matsuo K, Hotta K, et al. Identification of epigenetic aberrant promoter methylation in serum DNA is useful for early detection of lung cancer. Clin. Cancer Res. 2005;11:1219–25.
23. Hsu HS, Wen CK, Tang YA, Lin RK, Li WY, Hsu WH, et al. Promoter hypermethylation is the predominant mechanism in hMLH1 and hMSH2 deregulation and is a poor prognostic factor in nonsmoking lung cancer. Clin. Cancer Res. 2005;11:5410–6.
24. Soria J-C, Lee H-Y, Lee JI, Wang L, Issa J-P, Kemp BL, et al. Lack of PTEN expression in non-small cell lung cancer could be related to promoter methylation. Clin. Cancer Res. 2002;8:1178–84.
25. Tsou JA, Shen LYC, Siegmund KD, Long TI, Laird PW, Seneviratne CK, et al. Distinct DNA methylation profiles in malignant mesothelioma, lung adenocarcinoma, and non-tumor lung. Lung Cancer. 2005;47:193–204.
26. Olaussen KA, Soria JC, Park YW, Kim HJ, Kim SH, Ro JY, et al. Assessing abnormal gene promoter methylation in paraffin-embedded sputum from patients with NSCLC. Eur. J. Cancer. 2005;41:2112–9.
27. De Smet C, Loriot A, Boon T. Promoter-dependent mechanism leading to selective hypomethylation within the 5' region of gene MAGE-A1 in tumor cells. Mol. Cell. Biol. 2004;24:4781–90.

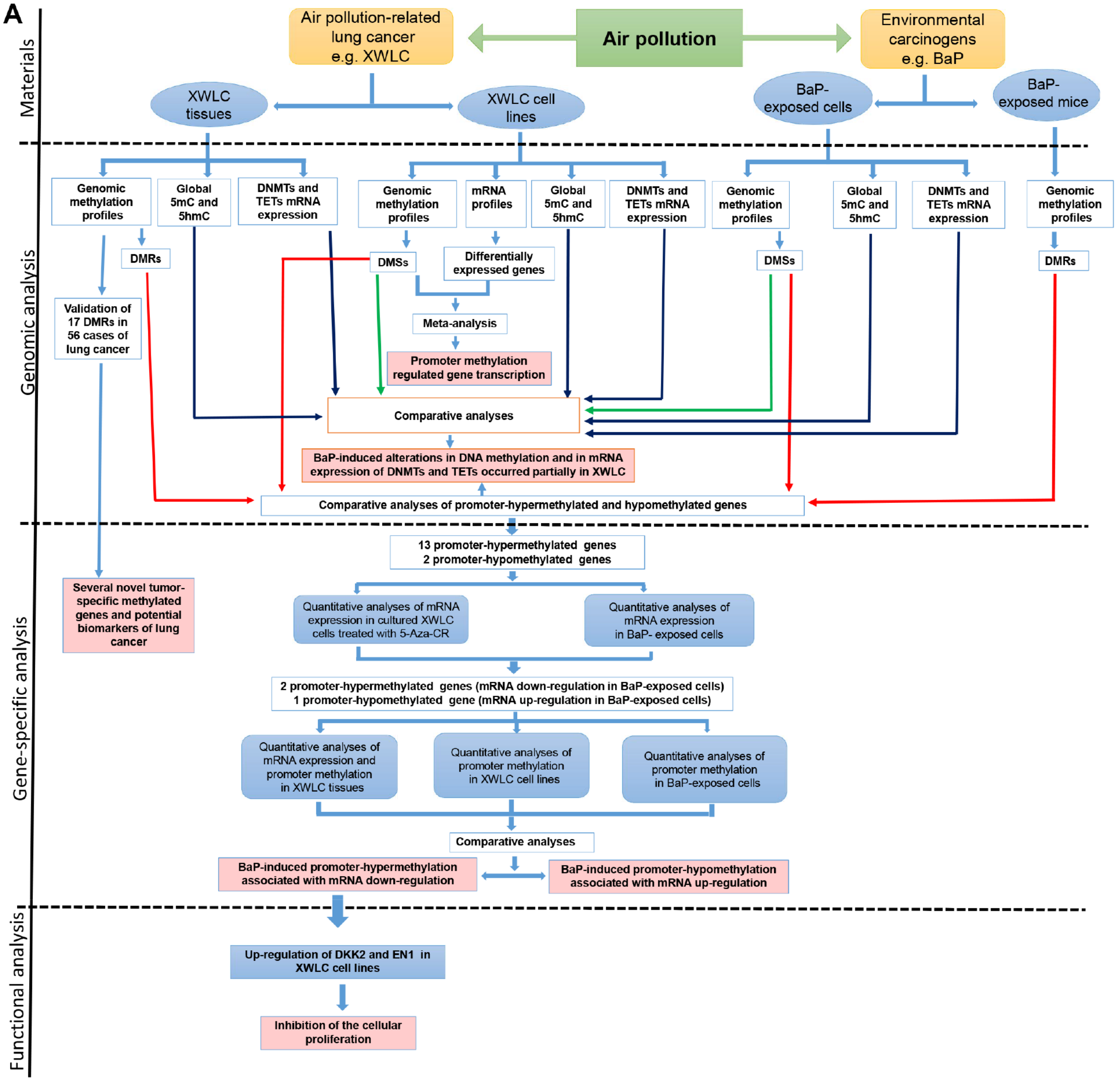
A
Materials
Genomic analysis
Gene-specific analysis
Functional analysis
Air pollution-related lung cancer e.g. XWLC
Air pollution
Environmental carcinogens e.g. BaP
XWLC tissues
XWLC cell lines
BaP-exposed cells
BaP-exposed mice
Genomic methylation profiles
Global 5mC and 5hmC
DNMTs and TETs mRNA expression
Genomic methylation profiles
mRNA profiles
Global 5mC and 5hmC
DNMTs and TETs mRNA expression
Genomic methylation profiles
Global 5mC and 5hmC
DNMTs and TETs mRNA expression
Genomic methylation profiles
DMRs
DMSs
Differentially expressed genes
DMSs
DMRs
Validation of 17 DMRs in 56 cases of lung cancer
Meta-analysis
Promoter methylation regulated gene transcription
Comparative analyses
BaP-induced alterations in DNA methylation and in mRNA expression of DNMTs and TETs occurred partially in XWLC
Comparative analyses of promoter-hypermethylated and hypomethylated genes
13 promoter-hypermethylated genes
2 promoter-hypomethylated genes
Several novel tumor-specific methylated genes and potential biomarkers of lung cancer
Quantitative analyses of mRNA expression in cultured XWLC cells treated with 5-Aza-CR
Quantitative analyses of mRNA expression in BaP- exposed cells
2 promoter-hypermethylated genes (mRNA down-regulation in BaP-exposed cells)
1 promoter-hypomethylated gene (mRNA up-regulation in BaP-exposed cells)
Quantitative analyses of mRNA expression and promoter methylation in XWLC tissues
Quantitative analyses of promoter methylation in XWLC cell lines
Quantitative analyses of promoter methylation in BaP-exposed cells
Comparative analyses
BaP-induced promoter-hypermethylation associated with mRNA down-regulation
BaP-induced promoter-hypomethylation associated with mRNA up-regulation
Up-regulation of DKK2 and EN1 in XWLC cell lines
Inhibition of the cellular proliferation

(*Continued*)

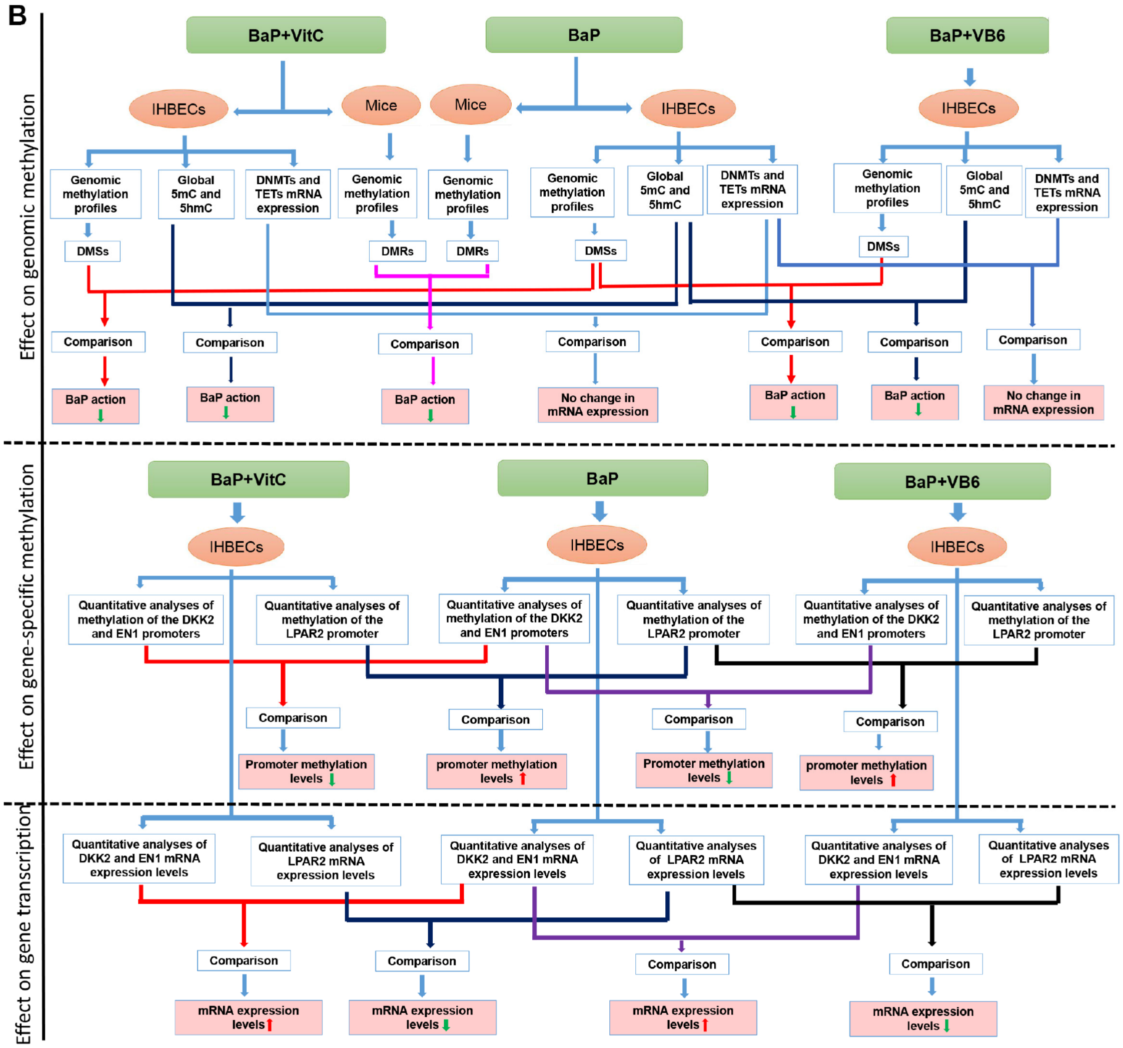

Supplementary Figure S9: Schematic diagram illustrating the study designs. A. analyses of genomic and gene-specific methylation and functional investigations; **B.** Studies of VitC and VB6 antagonism.

Supplementary Table S1: Information about the 65 lung cancer patients and cell lines used in this study

Supplementary Table S1A: Information about the 65 lung cancer patients used in this study

Supplementary Table S1B: Information about cell lines used in this study

See Supplementary File 1

Supplementary Table S2: Differentially methylated regions (DMRs) with a peak score ≥ 2 identified in the 14 XWLC and paracancerous tissues by CpG-methylation microarrays

See Supplementary File 2

Supplementary Table S3: Differentially promoter-methylated genes with |log2 ratio|> 1 in the 14 XWLC tissues

See Supplementary File 3

Supplementary Table S4: Relationships between the methylation statuses of the 17 selected genes and the characteristics of the 56 lung cancer patients

Gene	Gender		Age, y		Smoking		Histological type		TNM stage		BaP exposure	
	Female (n=26)	Male (n=30)	<60 (n=34)	≧60 (n=22)	No (n=28)	Yes (n=28)	ADC (n=46)	SCC (n=10)	I + II (n=32)	III+IV (n=24)	A (n=22)	B+C (n=34)
EN1	20(77)	23(77)[b]	23(68)	20(91)[b]*	21(75)	22(79)[b]	35(76)	8(80)[a]	26(81)	17(70)[b]	19(86)	24(71)[b]
HOXD10	19(73)	25(83)[b]	27(79)	17(77)[b]	22(79)	22(79)[b]	35(76)	9(90)[a]	24(75)	20(83)[b]	20(91)	24(71)[a]
IRX4	20(77)	21(70)[b]	25(74)	16(73)[b]	22(79)	19(68)[b]	33(72)	8(80)[a]	22(69)	19(79)[b]	19(86)	22(65)[b]
MEOX2	21(81)	20(67)[b]	24(71)	17(77)[b]	24(87)	17(61)[b]*	36(78)	5(50)[a]	35(78)	16(66)[b]	17(77)	24(71)[b]
NRN1	25(96)	25(83)[a]	30(88)	20(91)[a]	26(93)	24(86)[a]	42(91)	8(80)[a]	28(88)	22(92)[a]	20(91)	30(88)[a]
PRDM14	24(92)	21(70)[b]*	27(79)	18(82)[a]	26(93)	19(68)[b]*	36(78)	9(90)[a]	25(78)	20(83)[a]	16(73)	29(85)[b]
COL11A1	15(58)	17(57)[b]	20(59)	12(55)[b]	18(64)	14(50)[b]	27(59)	5(50)[a]	18(56)	14(58)[b]	15(68)	17(50)[b]
RELN	12(46)	12(40)[b]	16(47)	8(36)[b]	13(46)	12(39)[b]	21(45)	3(30)[a]	14(43)	10(41)[b]	12(55)	12(35)[b]
LRFN5	18(69)	19(63)[b]	22(65)	15(68)[b]	21(75)	16(57)[b]	31(67)	6(60)[a]	18(56)	19(79)[b]	18(82)	19(56)[b]*
NID2	16(62)	23(77)[b]	23(68)	16(73)[b]	20(71)	19(68)[b]	30(65)	9(90)[a]	21(66)	18(75)[b]	17(77)	22(65)[b]
NLGN4X	22(85)	12(40)[b]**	20(59)	14(64)[b]	12(86)	10(36)[b]**	31(67)	3(30)[a]*	31(66)	13(54)[b]	13(59)	21(62)[b]
SPON1	11(42)	10(33)[b]	14(41)	7(32)[b]	12(43)	9(32)[b]	17(37)	4(40)[a]	11(34)	10(42)[b]	12(55)	9(26)[b]*
ADCY8	13(50)	15(50)[b]	18(53)	10(45)[b]	16(57)	12(43)[b]	23(50)	5(50)[b]	15(47)	13(54)[b]	14(64)	14(41)[b]
ADRB3	12(46)	16(53)[b]	17(50)	11(50)[b]	13(46)	15(54)[b]	23(50)	5(50)[b]	14(44)	14(58)[b]	11(50)	17(50)[b]
RYR3	14(54)	14(47)[b]	17(50)	11(50)[b]	16(57)	12(43)[b]	24(52)	4(40)[b]	18(56)	10(42)[b]	13(59)	15(44)[b]
GRM8	16(62)	11(37)[b]	13(38)	14(64)[b]	17(61)	10(36)[b]	24(52)	3(30)[a]	17(53)	10(42)[b]	11(50)	16(47)[b]
YTHDF3	19(73)	20(67)[b]	24(71)	15(68)[b]	20(71)	19(68)[b]	32(70)	7(70)[a]	19(59)	20(83)[b]*	16(73)	23(68)[b]

The results are expressed as n (%)

a, Fisher's exact probability test; b, Chi-square test. $^{**}P \leq 0.01$ shows significant distinct difference, $^{*}P \leq 0.05$ shows distinct difference.

Supplementary Table S5: Differentially methylated sites (DMSs) with |beta difference| > 0.2 which occurred within gene 5'UTR, 1st exon, TSS200, TSS1500 and 3'UTR regions in lung cancer cell lines

See Supplementary File 4

Supplementary Table S6: Differentially methylated sites (DMSs) which occurred within gene 5'UTR, 1st exon, TSS200, TSS1500 and 3'UTR regions identified in BaP-exposed IHBECs

See Supplementary File 5

Supplementary Table S7: Differentially methylated regions (DMRs) identified in BaP-exposed murine tissues by MeDIP-Seq

See Supplementary File 6

Supplementary Table S8: Comparison of differentially methylated sites (DMSs) between lung cancer cell lines and IHBECs treated by 0.1 nM BaP for 24 days

See Supplementary File 7

Supplementary Table S9: Differentially expressed genes identified in lung cancer cell lines by gene expression microarrays

See Supplementary File 8

Supplementary Table S10: Comparison of hypermethylated and hypomethylated genes among XWLC tissues, XWLC cell lines, BaP-exposed IHBECs, and BaP-exposed murine tissues

See Supplementary File 9

Supplementary Table S11: Comparison of differentially methylated sites (DMSs) which occurred within gene 5'UTR, 1st exon, TSS200, TSS1500, body and 3'UTR regions in IHBECs between BaP plus VitC and BaP alone treatments

See Supplementary File 10

Supplementary Table S12: Comparison of differentially methylated sites (DMSs) which occurred within gene 5'UTR, 1st exon, TSS200, TSS1500, body and 3'UTR regions in IHBECs between BaP plus VB6 and BaP alone treatments

See Supplementary File 11

Supplementary Table 13: Comparison of differentially methylated regions (DMRs) in murine tissues treated either with BaP alone or with BaP plus VitC treatments

See Supplementary File 12

Supplementary Table S14: Lack of mutations in the TP53, KRAS, and EGFR genes in BaP-exposed 16HBE cells and murine tissues

Samples		TP53	KRAS	EGFR
**BaP-exposed 16HBE cells**		**exon2-11**	**exon2-5**	**exon18-21**
8 d	0.1nM BaP	-	-	-
	1nM BaP	-	-	-
24 d	0.1nM BaP	-	-	-
	1nM BaP	-	-	-
48 d	0.1nM BaP	-	-	-
	1nM BaP	-	-	-
**BaP-exposed murine tissues exon5-8**				
180 d	5nM BaP (3 cases)	-		
	50nM BaP (3 cases)	-		

Supplementary Table S15: Information for the primers used in this study

Supplementary Table S15A: Primer sequences used for MSP of 17 genes

Supplementary Table S15B: Primer sequences used for qRT-PCR

Supplementary Table S15C: Primer sequences used for BSP

Supplementary Table S15D: Primer sequences used for overexpression

Supplementary Table S15E: Primer sequences used for exon sequence

See Supplementary File 13
