# Supplementary material for: Air pollution and DNA methylation alterations in lung cancer: A systematic and comparative study
Source: Oncotarget. 2016 Nov 25;8(1):1369–91. doi: 10.18632/oncotarget.13622 (PMC5352062; doi:10.18632/oncotarget.13622)
Supplement: Supplementary file 13 [file oncotarget-08-1369-s013.docx]

**Supplementary Table 15:** Information for the primers used in this study.

**Supplementary Table 15A:** Primer sequences used for MSP of 17 genes.

| **Gene symbol** | **Primer Sequences 5’ to 3’** | **Annealing Temperature（℃）** | **Amplicon size** |
| --- | --- | --- | --- |
|  |  |  |  |
| ADCY8 | MF: TTAGGGAAGCGTCGTCGC |  | 144bp |
|  | MR: CCGAAAACGTAACTTAACGCG |  |  |
|  | UF: GGATTTAGGGAAGTGTTGTTGT | 56 | 152 bp |
|  | UR: AATTCCAAAAACATAACTTAACACA |  |  |
| ADRB3 | MF: CGTTTGGTGATTAGTGCGTC | 58 | 193 bp |
|  | MR: TCCTAATAATACCGCCGACG |  |  |
|  | UF: GTAGTGTTTGGTGATTAGTGTGTT | 54 | 201 bp |
|  | UR: AAACTCCTAATAATACCACCAACA |  |  |
| RYR3 | MF: GTTGGTATTTAGTTTGGGCGC | 62 | 114 bp |
|  | MR: CACACTCAACGAAAACGCG |  |  |
|  | UF: GGGTTGGTATTTAGTTTGGGTGT | 61 | 119 bp |
|  | UR: CCACACACTCAACAAAAACACA |  |  |
| COL11A1 | MF: AAATTTGATGGTTTGCGTTTTTC | 62 | 97 bp |
|  | MR: AAATCAAAAAATCGCGACCG |  |  |
|  | UF: AAATTTGATGGTTTGTGTTTTTT | 58 | 99 bp |
|  | UR: AAAAATCAAAAAATCACAACCA |  |  |
| LRFN5 | MF: TGCGTAGTTTTTTAGCGGTTC | 58 | 120bp |
|  | MR: CTCCAAATCGACGAACGACG |  |  |
|  | UF: TTTGTGTAGTTTTTTAGTGGTTT | 58 | 124bp |
|  | UR: CCCTCCAAATCAACAAACAACA |  |  |
| NID2 | MF: GTTTAAGGACGTTATTTTTTCGC | 56 | 153bp |
|  | MR: CCTAAACCTACTACAACGCG |  |  |
|  | UF: TAAGTTTAAGGATGTTATTTTTTTGT | 56 | 157bp |
|  | UR: CCCTAAACCTACTACAACACA |  |  |
| NLGN4X | MF: GTTGTGGGCGTTTTAGTCGC | 64 | 133 bp |
|  | MR: AAAAATCCGCCAAACGCG |  |  |
|  | UF: GGGTTGTGGGTGTTTTAGTTGT | 60 | 142 bp |
|  | UR: TAATAAAAAAAATCCACCAAACACA |  |  |
| RELN | MF: GGGGTAATAGTTAGTCGTCGC | 61 | 147 bp |
|  | MR: CTAAATAAACGAACGACGCG |  |  |
|  | UF: GGGGTAATAGTTAGTTGTTGTGT | 56 | 152 bp |
|  | UR: CAAAACTAAATAAACAAACAACACA |  |  |
| SPON1 | MF: GGTTATTGTAGTCGTATTTTGCGC | 62 | 129 bp |
|  | MR: GAACGACCACTTACCGCG |  |  |
|  | UF: GGGTTATTGTAGTTGTATTTTGTGT | 57 | 132 bp |
|  | UR: CCAAACAACCACTTACCACA |  |  |
| EN1 | MF: TTTGGTAGTGGGAGTTCGGGTAAC | 65 | 86 bp |
|  | MR: AAACCTAACGCGCATCCGCG |  |  |
|  | UF: TTTTTGGTAGTGGGAGTTTGGGTAAT | 58 | 91bp |
|  | UR: CTAAAACCTAACACACATCCACA |  |  |
| HOXD10 | MF: GTTTGGTTTTTCGGTTTTCGC | 64 | 141bp |
|  | MR: GAAAAAATAAATCGAATTCCGCG |  |  |
|  | UF: TTGTTTGGTTTTTTGGTTTTTGT | 58 | 145bp |
|  | UR: ACAAAAAAATAAATCAAATTCCACA |  |  |
| IRX4 | MF: GCGATTGTGAGTATTTCGTC | 55 | 159 bp |
|  | MR: CTCCAATTAAACCCCGCCG |  |  |
|  | UF: TTTTGGTGATTGTGAGTATTTTGTT | 55 | 165 bp |
|  | UR: ACTCCAATTAAACCCCACCA |  |  |
| MEOX2 | MF: ACGGCGTAAGGTTTGTATTC | 62 | 124bp |
|  | MR: TCTTCGTTAAAATATCCCGCG |  |  |
|  | UF: GTTATGGTGTAAGGTTTGTATTT | 58 | 124bp |
|  | UR: CCTCTTCATTAAAATATCCCACA |  |  |
| NRN1 | MF: TCGTTTATATTTCGGACGGTAGC | 62 | 94 bp |
|  | MR: AAAAACCCCGAACCGCG |  |  |
|  | UF: AGTTTGTTTATATTTTGGATGGTAGT | 61 | 98 bp |
|  | UR: CAAAAACCCCAAACCACA |  |  |
| PRDM14 | MF: TTTAGCGGTGTGATTTCGC | 58 | 123 bp |
|  | MR: TAACGAACGCTACCCGCG |  |  |
|  | UF: TTGGTTTAGTGGTGTGATTTTGT | 58 | 134 bp |
|  | UR: AAAAATATAACAAACACTACCCACA |  |  |
| GRM8 | MF: CGAAGGTATTTCGTTATTTCGGC | 58 | 120bp |
|  | MR: TACGACGACAAAATTTTAAAACG |  |  |
|  | UF: TGAAGGTATTTTGTTATTTTGGT | 58 | 120bp |
|  | UR: CATACAACAACAAAATTTTAAAACA |  |  |
| YTHDF3 | MF: TCGTGAAAAGAGTCGTCGC | 61 | 127 bp |
|  | MR: AAAACGCCTAAACCGAACG |  |  |
|  | UF: TGTTGTGAAAAGAGTTGTTGT | 57 | 131 bp |
|  | UR: TAAAAACACCTAAACCAAACA |  |  |

MF: Methylation Primer Sequence Forward; MR: Methylation Primer Sequence Reverse; UF: Unmethylation Primer Sequence Forward; UR: Unmethylation Primer Sequence Reverse

**Supplementary Table 15B:** Primer sequences used for qRT-PCR

| **Gene symbol** | **Primer Sequences 5’ to 3’** | **Amplicon size（bp）** |
| --- | --- | --- |
| DNMT1 | F：AAGCCGTCAAGACTGATGGG | 112 |
|  | R：GGTTTTGAGGAATCATCTGGAATA |  |
| DNMT3A | F：TGATGGAATCGCTACAGGGC | 293 |
|  | R：GGAGGCGGTAGAACTCAAAGAA |  |
| DNMT3B | F：TGGCACTGGGGCTGTTCA | 105 |
|  | R：CTCGCACCCTAGCTTTCTCC |  |
| TET1 | F：ATACAATGGGCACCCTACCG | 159 |
|  | R：GGGCTTGGGCTTCTACCAAA |  |
| TET2 | F：GCTGACAAACTCTACTCGG | 188 |
|  | R：CTTCTGGCAAACTTACATCC |  |
| TET3 | F：CCCAAAGAGGAAGAAGTG | 129 |
|  | R：GCAGTCAATCGCTATTTC |  |
| DKK2 | F：CTCACAGATCGGCAGTTCG | 113 |
|  | R：ATGCCAGTCCTTGGTACATGC |  |
| LRRC33 | F：CAAGACCCTGTGGAATCACTC | 164 |
|  | R：TCTCTTCGTAGTTCTCTGAGAGG |  |
| GATA2 | F：ACTGACGGAGAGCATGAAGAT | 116 |
|  | R：CCGGCACATAGGAGGGGTA |  |
| PRDM12 | F：GGGAGGTGTTCAATGAGGATG | 111 |
|  | R：TCTGCTCCTGTTCGTTACGTG |  |
| PTGDR | F：GGTGCTTTATCCAGATGGTCC | 143 |
|  | R：GTGCATCGCATAGAGGTTGC |  |
| HOXA11 | F：TGCCAAGTTGTACTTACTACGTC | 107 |
|  | R：GTTGGAGGAGTAGGAGTATGTCA |  |
| DCTN1 | F：GACTGAAACGGGCAGAAGACA | 185 |
|  | R：CTCCTCCATATAGCGTTCCTTTG |  |
| SPHKAP | F：TCTGCTTGGTCCAATGTGCAA | 158 |
|  | R：GGAACAGTTCGTGTCATCCTC |  |
| ADCYAP1 | F：CCACTCGGACGGGATCTTC | 75 |
|  | R：GCCGCCAAGTATTTCTTGACAG |  |
| SALL1 | F：CAACGTCATCATCGAGAACCTC | 129 |
|  | R：AGAGCTAGGAGTTGTTCCATGAG |  |
| EN1 | F：GAGCGCAGGGCACCAAATA | 92 |
|  | R：CGAGTCAGTTTTGACCACGG |  |
| HS3ST2 | F：CCTCTCCGGTTCCAACCAC | 119 |
|  | R：GTACTCGGATAAACTCCAGCAC |  |
| HTR1B | F：AACTACCTGATCGCCTCTCTG | 144 |
|  | R：ACAAGTGATGTCCGACGACAG |  |
| LPAR2 | F：CCTGGTCAAGACTGTTGTCATC | 96 |
|  | R：GACTCACAGCCTAAACCATCC |  |
| MAGEA1 | F：CCATCAACTTCACTCGACAGAG | 97 |
|  | R：CGGAACAAGGACTCCAGGATA |  |
| GAPDH | F：GTTGCCATCAATGACCCCTT | 201 |
|  | R：CTCCACGACGTACTCAGCG |  |

**Supplementary Table 15C:** Primer sequences used for BSP

| **Gene symbol** | **Primer Sequences 5’ to 3’** | **Amplicon size（bp）** |
| --- | --- | --- |
| EN1 | F：TTTTGGTTAGTTGAAGTTGTGA | 387 |
|  | R：AAAACTCTAAAATAACACCCCC |  |
| DKK2 | F：GGAAAGGGGTAGATTAAAGAGG | 495 |
|  | R：ACTTAACCACCAAACATCACC |  |
| LPAR2 | F：GTTTGYGGGGTTGAGTTA | 238 |
|  | R：TACTACRAACTAACAAAACCRAAAC |  |

**Supplementary Table 15D:** Primer sequences used for overexpression

| **Gene symbol** | **Primer Sequences 5’ to 3’** | |
| --- | --- | --- |
| EN1 | F- Xba I | GCTCTAGAGCCACCATGGAAGAACAGCAGCCGGAACCT |
|  | R-EcoR I | CCGGAATTCCTACTCGCTCTCGTCTTTGTCCTGGA |
| DKK2 | F-EcoR I | CCGGAATTCGCCACCATGGCCGCGTTGATGCGGAGCAAG |
|  | R-Not I | AAGGAAAAAAGCGGCCGCTCAAATTTTCTGACACACATGG |

**Supplementary Table 15E:** Primer sequences used for exon sequence

|  | **Gene-Exon** | **Primer Sequences 5’ to 3’** |
| --- | --- | --- |
| Human | TP53-Exon2 | F: GGAAGTGTCTCATGCTGGA |
|  |  | R: CAGAACGTTGTTTTCAGGAA |
|  | TP53-Exon3 | F: AGCGAAAATTCCATGGGACT |
|  |  | R: TCCATTGCTTGGGACGGCAA |
|  | TP53-Exon4 | F: CAGAGATCACACATTAAGTGGGTAA |
|  |  | R: GGACTGACTTTCTGCTCTTGTCTTT |
|  | TP53-Exon5 | F: ATGGGGTTATAGGGAGGTCAAAT |
|  |  | R: AACTCTCTCTAGCTCGCTAGTGG |
|  | TP53-Exon6 | F: TGGGGTTATAGGGAGGTCAAA |
|  |  | R: GCCATGGCCATCTACAAGCA |
|  | TP53-Exon7 | F: GCCACAGGTCTCCCCAAGGC |
|  |  | R: GATGGGTAGTAGTATGGAAGAA |
|  | TP53-Exon8, 9 | F: GTCATAGAACCATTTTCATGCTCTC |
|  |  | R: CTTCCATACTACTACCCATCCACCT |
|  | TP53-Exon10 | F: ATACACTGAGGCAAGAATGTGGTTA |
|  |  | R: CAGTTTCTACTAAATGCATGTTGCT |
|  | TP53-Exon11 | F: GTATGTCCTACTCCCCATCCTCCT |
|  |  | R: ACCATCTTGATTTGAATTCCCGTT |
|  | EGFR-Exon17 | F: GGCGTGGAAACAGACATAGAA |
|  |  | R: TGGAGTTTCCCAAACACTCAG |
|  | EGFR-Exon18 | F: ATTCGTGGAGCCCAACAG |
|  |  | R: GCCAGTAATTGCCTGTTTCC |
|  | EGFR-Exon19 | F: CTCTCCCACTGCATCTGTCA |
|  |  | R: GATGGGACAGGCACTGATT |
|  | EGFR-Exon20 | F: GTCAGCAGCGGGTTACATCT |
|  |  | R: AAGCAGCTCTGGCTCACACT |
|  | KRAS-Exon2 | F: CAATACCAAGAAACCCATAAAAATAA |
|  |  | R: GGTCCACTAGGAAAACTGTAACAATA |
|  | KRAS-Exon3 | F: AAGTTACTCCACTGCTCTAATCCCCC |
|  |  | R: ATCCAGACTGTGTTTCTCCCTTCTCA |
|  | KRAS-Exon4 | F: AGCAGTACCATGGACACTGGATTA |
|  |  | R: AAGTTGTGGACAGGTTTTGAAAGA |
|  | KRAS-Exon5 | F: CCTAGAAGAATCATCATCAGGAAGCC |
|  |  | R: GCCAATGTGGAAAAAATAGTAGCAAA |
| Mouse | *Trp53*-Exon5 | F: AGGGCGTCCAATGGTGCT |
|  |  | R: GGCGGTGTTGAGGGCTTA |
|  | *Trp53*-Exon6 | F: ACTGGCAGCCTCCCATCTC |
|  |  | R: CACGGCAGCTTGCACCTCT |
|  | *Trp53*-Exon7 | F: CTGTAGTGAGGTAGGGAGC |
|  |  | R: CAGAGGAGGAGACTTCATTTA |
|  | *Trp53*-Exon8 | F: TACTGCCTTGTGCTGGTCCTTTTC |
|  |  | R: AAGAGGTGACTTTGGGGTGAAGCTC |
