# Supplementary material for: Air pollution and DNA methylation alterations in lung cancer: A systematic and comparative study
Source: Oncotarget. 2016 Nov 25;8(1):1369–91. doi: 10.18632/oncotarget.13622 (PMC5352062; doi:10.18632/oncotarget.13622)
Supplement: Supplementary file 14 [file oncotarget-08-1369-s014.doc]

**Supplementary Table 1:** Information about the 65 lung cancer patients and cell lines used in this study.

**Supplementary Table 1A:** Information about the 65 lung cancer patients used in this study.

| **Number** | **Gender** | **Age-yr** | **Residence** | **Smoking status** | **Histology subtype** | **TNM stage** | **BaP exposure** | **DNA methylation and mRNA expression** |
| --- | --- | --- | --- | --- | --- | --- | --- | --- |
| 1 | FM | 62 | 1 | NO | AD | ⅠB | A | Microarray/MSP |
| 2 | M | 38 | 2 | YES | SCC | ⅢB | B | Microarray/MSP/BSP/qPCR |
| 3 | FM | 42 | 1 | NO | AD | ⅠA | B | Microarray/MSP/BSP/qPCR |
| 4 | M | 51 | 1 | YES | AD | ⅠB | A | Microarray/MSP/BSP/qPCR |
| 5 | FM | 42 | 2 | NO | AD | ⅠA | B | Microarray/MSP |
| 6 | M | 64 | 2 | YES | AD | ⅠA | B | Microarray/MSP |
| 7 | FM | 52 | 2 | NO | AD | ⅠB | A | Microarray/MSP |
| 8 | M | 49 | 2 | YES | AD | Ⅳ | C | Microarray/MSP |
| 9 | FM | 60 | 2 | NO | AD | ⅠB | C | Microarray/MSP/BSP/qPCR |
| 10 | M | 60 | 1 | YES | SCC | ⅢB | A | Microarray/MSP |
| 11 | FM | 53 | 2 | NO | AD | ⅠB | A | Microarray |
| 12 | FM | 56 | 2 | NO | AD | ⅡB | A | Microarray/MSP |
| 13 | M | 61 | 2 | YES | SCC | ⅢA | C | Microarray |
| 14 | FM | 59 | 2 | NO | AD | ⅠA | A | Microarray |
| 15 | M | 43 | 2 | YES | AD | ⅡB | C | MSP |
| 16 | M | 66 | 3 | NO | AD | ⅠB |  | MSP |
| 17 | M | 49 | 1 | YES | SCC | ⅢA | A | MSP |
| 18 | M | 76 | 1 | YES | SCC | ⅠB | A | MSP |
| 19 | M | 62 | 2 | YES | AD | Ⅳ | B | MSP |
| 20 | FM | 62 | 2 | YES | AD | ⅡB | B | MSP |
| 21 | FM | 69 | 2 | NO | SCC | ⅢA | C | MSP |
| 22 | M | 66 | 2 | YES | AD | ⅠB | C | MSP |
| 23 | M | 64 | 3 | YES | SCC | ⅡB |  | MSP |
| 24 | M | 63 | 3 | YES | SCC | ⅡB |  | MSP |
| 25 | FM | 42 | 2 | NO | AD | ⅠA | B | MSP |
| 26 | FM | 47 | 2 | NO | AD | Ⅳ | B | MSP |
| 27 | M | 58 | 2 | YES | AD | ⅢA | C | MSP |
| 28 | M | 61 | 2 | YES | AD | ⅡB | B | MSP |
| 29 | FM | 41 | 2 | NO | AD | ⅢB | A | MSP |
| 30 | FM | 57 | 2 | NO | AD | ⅢA | B | MSP |
| 31 | FM | 56 | 2 | NO | AD | ⅠB | A | MSP |
| 32 | M | 42 | 2 | YES | AD | ⅡB | A | MSP |
| 33 | M | 44 | 2 | YES | AD | ⅠA | C | MSP |
| 34 | M | 56 | 3 | YES | SCC | ⅡB |  | MSP |
| 35 | M | 44 | 2 | YES | AD | Ⅳ | A | MSP |
| 36 | M | 70 | 2 | NO | AD | ⅢB | C | MSP |
| 37 | FM | 40 | 2 | NO | AD | ⅠB | C | MSP |
| 38 | M | 57 | 2 | YES | AD | ⅢA | A | MSP |
| 39 | M | 62 | 2 | YES | AD | ⅡA | A | MSP |
| 40 | FM | 54 | 2 | NO | AD | ⅡB | A | MSP |
| 41 | FM | 62 | 2 | NO | AD | ⅢA | B | MSP |
| 42 | FM | 70 | 3 | NO | AD | ⅡB |  | MSP |
| 43 | M | 59 | 3 | NO | AD | ⅢB |  | MSP |
| 44 | M | 47 | 1 | NO | AD | ⅢA | A | MSP |
| 45 | FM | 36 | 2 | NO | AD | ⅠB | B | MSP |
| 46 | FM | 60 | 2 | NO | AD | ⅠB | A | MSP |
| 47 | M | 59 | 3 | YES | SCC | ⅢB |  | MSP |
| 48 | M | 59 | 2 | YES | AD | ⅢA | A | MSP |
| 49 | FM | 48 | 2 | NO | AD | ⅠB | B | MSP |
| 50 | FM | 68 | 2 | NO | AD | ⅢB | A | MSP |
| 51 | FM | 48 | 2 | NO | AD | ⅢB | B | MSP |
| 52 | FM | 50 | 1 | NO | AD | ⅠB | B | MSP |
| 53 | FM | 55 | 2 | NO | AD | ⅠA | C | MSP |
| 54 | FM | 57 | 2 | YES | AD | ⅢA | C | MSP |
| 55 | M | 39 | 1 | YES | AD | ⅢA | A | MSP |
| 56 | M | 64 | 2 | YES | AD | ⅠB | A | MSP |
| 57 | FM | 65 | 1 | NO | SCC | ⅢA | A | MSP |
| 58 | M | 41 | 2 | YES | AD | Ⅳ | B | MSP/BSP/qPCR |
| 59 | M | 69 | 1 | YES | AD | ⅡB | A | MSP/BSP/qPCR |
| 60 | FM | 63 | 1 | NO | SCC |  | A | BSP/qPCR |
| 61 | FM | 41 | 1 | NO | AD |  | A | BSP/qPCR |
| 62 | M | 54 | 1 | YES | SCC |  | A | BSP/qPCR |
| 63 | M | 53 | 1 | YES | AD |  | A | BSP/qPCR |
| 64 | M | 55 | 1 | NO | SCC |  | B | BSP/qPCR |
| 65 | FM | 48 | 1 | NO | AD |  | A | BSP/qPCR |

In Residence, 1 and 2 represent XW or FY in Yunnan, 3 represent non -XW or FY in Yunnan.

In BaP exposure, A, B and C represents high, Medium and low degrees of BaP exposure, respectively.

**Supplementary Table 1B:** Information about cell lines used in this study.

| **Cell type/ name** | **Culture media** | **Resource** |
| --- | --- | --- |
| **Immortalized human bronchial epithelial cell lines** | | |
| 16HBE | DMEM 10%FBS | Institute of Zoology, CAS, Beijing, China |
| HBEpiC | BEpiCM | Institute of Zoology, CAS, Beijing, China |
| **Lung adenocarcinoma** | | |
| XLA-07 | RPMI 10%FBS | Kunming Medical University, Kunming, China |
| XL-JT | RPMI 10%FBS | Kunming Medical University, Kunming, China |
| A549 | RPMI 10%FBS | American Type Culture Collection |
| **Lung squamous carcinoma** | | |
| EPLC-32M1 | RPMI 10%FBS | German Cancer Research Center, Heidelberg, Germany |
| **Human embryonic kidney cells** | | |
| 293TN cells | DMEM 10%FBS | American Type Culture Collection |
